# Expression pattern analysis of m6A regulators reveals IGF2BP3 as a key modulator in osteoarthritis synovial macrophages

**DOI:** 10.1186/s12967-023-04173-9

**Published:** 2023-05-22

**Authors:** Yuheng Lu, Hongbo Zhang, Haoyan Pan, Zhicheng Zhang, Hua Zeng, Haoyu Xie, Jianbin Yin, Wen Tang, Rengui Lin, Chun Zeng, Daozhang Cai

**Affiliations:** 1 Department of Orthopedics, Academy of Orthopedics, Guangdong Province, Guangdong Provincial Key Laboratory of Bone and Joint Degeneration Diseases, The Third Affiliated Hospital of Southern Medical University, Guangzhou, 510515 China; 2 Department of Joint Surgery, Center for Orthopedic Surgery, Orthopedic Hospital of Guangdong Province, The Third Affiliated Hospital of Southern Medical University, Guangzhou, China; 3 The Third School of Clinical Medicine, Southern Medical University, Guangzhou, China

**Keywords:** m6A regulators, IGF2BP3, Osteoarthritis, Synovial macrophages

## Abstract

**Background:**

Disruption of N6 methyl adenosine (m6A) modulation hampers gene expression and cellular functions, leading to various illnesses. However, the role of m6A modification in osteoarthritis (OA) synovitis remains unclear. This study aimed to explore the expression patterns of m6A regulators in OA synovial cell clusters and identify key m6A regulators that mediate synovial macrophage phenotypes.

**Methods:**

The expression patterns of m6A regulators in the OA synovium were illustrated by analyzing bulk RNA-seq data. Next, we built an OA LASSO-Cox regression prediction model to identify the core m6A regulators. Potential target genes of these m6A regulators were identified by analyzing data from the RM2target database. A molecular functional network based on core m6A regulators and their target genes was constructed using the STRING database. Single-cell RNA-seq data were collected to verify the effects of m6A regulators on synovial cell clusters. Conjoint analyses of bulk and single-cell RNA-seq data were performed to validate the correlation between m6A regulators, synovial clusters, and disease conditions. After IGF2BP3 was screened as a potential modulator in OA macrophages, the IGF2BP3 expression level was tested in OA synovium and macrophages, and its functions were further tested by overexpression and knockdown in vitro.

**Results:**

OA synovium showed aberrant expression patterns of m6A regulators. Based on these regulators, we constructed a well-fitting OA prediction model comprising six factors (FTO, YTHDC1, METTL5, IGF2BP3, ZC3H13, and HNRNPC). The functional network indicated that these factors were closely associated with OA synovial phenotypic alterations. Among these regulators, the m6A reader IGF2BP3 was identified as a potential macrophage mediator. Finally, IGF2BP3 upregulation was verified in the OA synovium, which promoted macrophage M1 polarization and inflammation.

**Conclusions:**

Our findings revealed the functions of m6A regulators in OA synovium and highlighted the association between IGF2BP3 and enhanced M1 polarization and inflammation in OA macrophages, providing novel molecular targets for OA diagnosis and treatment.

**Supplementary Information:**

The online version contains supplementary material available at 10.1186/s12967-023-04173-9.

## Background

Osteoarthritis (OA) is the most common disabling joint disease that seriously hampers the quality of life. By 2020, OA is expected to affect over 500 million people worldwide [1]. As a multifactorial disease, mechanical overloading, trauma, inflammation, metabolism, and genetic vulnerabilities seem to be the potential risk factors of OA [2]. However, given its unclear pathogenesis, no radical cure for OA has been discovered. Current therapeutic strategies focus on pain relief and lubrication, whereas knee replacement is the only option for patients with late-stage OA to partially gain motor function [3]. Therefore, elucidating the mechanisms of OA is important for disease prevention and eradication.

As a whole-joint disease, pathological changes in OA involve a wide range of articular tissues, including cartilage, subchondral bone, ligaments, menisci, fat pads, and synovium [4]. The synovium exhibits abnormalities at the onset of OA, even before visible cartilage loss, and the degree of synovitis is closely related to disease progression [5]. As major immunocytes in the synovium, emerging evidence suggests that synovial macrophages play a pivotal role in OA development [6]. In brief, OA macrophages polarized to the proinflammatory M1 subtype marked by CD86 (or iNOS in mice), which secrete inflammatory cytokines (such as IL-1β, IL-6, and TNF-α) to accelerate synovitis and chondrocyte senescence [7]. While the M2 polarization of macrophages, marked by CD206 (also named MRC1), is largely inhibited, leading to decreased production of anti-inflammatory mediators (such as IL-4 and IL-10) and insufficient tissue repair [8]. Importantly, studies have claimed that cleansing of pathological macrophages relieves joint pain, synovitis, cartilage damage, and osteophyte formation [9, 10]. Therefore, targeting synovial macrophages may be a promising approach for treating OA.

N6-methyladenosine (m6A) modification is the most prevalent post-transcriptional modification in mammals, occurring in nearly 0.1–0.4% of adenosines, accounting for approximately 50% of all methylated ribonucleosides [11]. Over 80% of m6A modifications appear in messenger RNA (mRNA) and are mainly detected in the consensus sequence RRACH (R = A or G and H = A, C, or U) near transcript termination codons and 3′ untranslated regions (3′ UTRs) [12, 13]. As a reversible process, the m6A modification is precisely controlled by three regulatory proteins: writers, erasers, and readers [14]. Writers are a group of methyltransferases that recognize RRACH sequences with a methyltransferase domain and transfer the methyl group from S-adenosylmethionine to the adenosine of RNA [15]. In contrast, erasers are primarily demethyltransferases that catalyze the removal of m6A from RNA [14]. After modulated by writers and erasers, readers with unique m6A recognition domains bind to m6A-modified mRNA and enhance its stability by binding with RNA stabilizers (such as HuR, matrin 3), or promote its decay by facilitating the bridge between mRNA and RNase P/endoribonucleases [16, 17]. Besides, m6A modification regulates other mRNA dynamic processes such as splicing, export, and translation, while these processes also interact closely with mRNA stability and decay [17]. Recent studies have found that aberrant m6A modifications may be correlated with OA [18–22]. Overexpression of the m6A writer METTL3 inhibited extracellular matrix (ECM) synthesis in chondrocytes, whereas ATG7 modified by METTL3 inhibited autophagy and promoted senescence in OA fibroblasts [18, 23]. FTO-dependent m6A demethylation mediates the upregulation of AC008 to induce OA chondrocyte apoptosis [22]. However, the mechanism by which m6A regulators participate in the pathogenesis of OA synovitis remains unclear.

Insulin-like growth factor 2 mRNA-binding protein 3 (IGF2BP3) is an important m6A reader in eukaryotes [24]. It preferentially binds to m6A-containing mRNAs and enhances their stability by protecting them from endonuclease digestion and miRNA-induced degradation [25]. IGF2BP3 is a well-known oncogene that promotes cancer cell proliferation, survival, drug resistance, and metastasis [26–28]. It is also identified as an inflammation-triggering factor by activating NF-κB signalling to enhance epithelial cell injury [29]. In OA, IGF2BP3 was found upregulated in destructed cartilage and IL-1β induced chondrocytes, while the expression and function of IGF2BP3 in OA synovitis is unclear [30].

This study aimed to explore the expression patterns of m6A regulators in OA synovial cell clusters and identify key m6A regulators that mediate synovial macrophage phenotypes. The levels of major m6A regulators and their potential targets in the OA synovium were obtained from bulk RNA-seq data. Next, the locations of m6A regulators and their downstream targets were matched to specific cell clusters by analyzing single-cell RNA-seq (scRNA-seq) data. Among all the core m6A regulators, the m6A reader IGF2BP3 was verified to be upregulated in OA synovial macrophages and to play an important role in promoting macrophage inflammation and M1 polarization. Our study showed that the expression patterns of m6A regulators differed significantly between normal and OA synovium, as well as among different synovial cell clusters. Targeted regulation of m6A regulators that are mainly expressed and take effect in OA synovial cell clusters may serve as a promising approach to modulate the functions of these cells, thus alleviating OA synovitis progression.

## Methods

### Clinical samples

Our study was approved by the Institutional Review Board (IRB) of the Third Affiliated Hospital of Southern Medical University (Ethics approval code: 2022-lunshen-053). All patients involved signed written informed consent. Synovial samples were collected from 10 late-stage OA patients undergoing total knee replacement surgery, and normal synovium samples were obtained from 10 patients during arthroscopy for trauma or joint derangements. Patients with hypertension, diabetes, hyperlipidemia, rheumatoid arthritis (RA), other diseases affecting joints, and body mass index (BMI) greater than 35 were excluded from this study. The overall characteristics including genders, ages, and BMI were listed in Table 1.

**Table 1:** Clinical characteristics of the study groups

	NC (n = 10)	OA (n = 10)	P value
Sex			1.000
Female	4 (40.0%)	5 (50.0%)	
Male	6 (60.0%)	5 (50.0%)	
Age	59.1 (4.72)	62.5 (6.24)	0.188
BMI	25.6 (3.34)	26.5 (2.51)	0.511

### Cells

Bone marrow derived macrophages (BMDMs) were harvested from bone marrow of 6-week-old male C57BL/6J mice. After the mice were sacrificed, femurs and tibias were separated and collected. Bone marrow cavities were exposed and flushed with complete DMEM (Gibco, Carlsbad, CA, USA) containing 10% fetal bovine serum (FBS) (Gibco). Red cells were then removed. The remaining cells were maintained for 24 h in complete DMEM. Non-adherent cells were centrifugally collected and planted in the complete DMEM containing 10% FBS. Meanwhile, 30 ng/mL macrophage colony stimulating factor (M-CSF, R&D systems, Minneapolis, MN, USA) was also supplemented to induce the survival, proliferation, and differentiation of macrophages for 72 h.

BMDMs administered with 500 ng/mL lipopolysaccharide (LPS) (Invitrogen, San Diego, CA, USA), 5 ng/mL interleukin-1β (IL-1β) (R&D systems) or 20 ng/mL IL-4 (R&D systems) were harvested 24 h after treatment. Lipofectamine 3000 (Thermo Fisher Scientific, Waltham, MA, USA) and Lipofectamine RNAiMAX (Thermo Fisher Scientific) were used for plasmids and siRNA transfection. BMDMs transfected with IGF2BP3 overexpressing plasmids (2ug/mL) (Tsingke, Beijing, China) or IGF2BP3-siRNA (Tsingke) were collected 48 h for RNA and 60 h for protein after transfection. As for mechanical overloading, we applied the methods that have been validated in our previous study [31]. In brief, BMDMs were seeded into silicon stretch chambers coated with fibronectin at a density of 1 × 10^5^ cells/chamber. Cyclic tensile strain of 20% elongation was applied for 24 h to induce mechanical overloading cell model by using FLEXCELL-5000 mechanical stretch system in a CO2 incubator. Control cells were seeded onto the same plate and cultured without cyclic tensile strain.

### The qRT-PCR

Total RNA was isolated from BMDMs grown in 6-well plates using 1 mL/well TRIzol reagent (Takara Bio Inc., Shiga, Japan). 1 μg RNA sample was reverse transcribed to produce cDNA with Reverse transcription kit (Vazyme Biotech, Nanjing, China). The quantitative PCR (qPCR) assays were conducted to testify the expression levels of IGF2BP3, iNOS, CD206, IL-1β, IL6, TNF-α, IL-4 and IL-10 mRNAs relative to GAPDH mRNA applying Real-Time PCR Mix (Vazyme Biotech) in a 2 × ChamQ SYBR qPCR Master Mix (Vazyme Biotech). Primers for qPCR in this study were listed in Table 2.

**Table 2:** Primers used in this study

Genes	Primers	Sequences
IGF2BP3	Forward	GATTCGGAAACGGCAGTTGTA
	Reverse	TGGGATGTAGGCAACCTTCAA
iNOS	Forward	GTTCTCAGCCCAACAATACAAGA
	Reverse	GTGGACGGGTCGATGTCAC
CD206	Forward	CTCTGTTCAGCTATTGGACGC
	Reverse	CGGAATTTCTGGGATTCAGCTTC
IL-1β	Forward	TTCAGGCAGGCAGTATCACTC
	Reverse	GAAGGTCCACGGGAAAGACAC
IL-6	Forward	TAGTCCTTCCTACCCCAATTTCC
	Reverse	TTGGTCCTTAGCCACTCCTTC
TNF-α	Forward	CCCTCACACTCAGATCATCTTCT
	Reverse	GCTACGACGTGGGCTACAG
IL-4	Forward	GGTCTCAACCCCCAGCTAGT
	Reverse	GCCGATGATCTCTCTCAAGTGAT
IL-10	Forward	GCTCTTACTGACTGGCATGAG
	Reverse	CGCAGCTCTAGGAGCATGTG

### Flow cytometry

BMDMs were treated with FcR Blocking Reagent (BioLegend), and incubated with PE F4/80 (BioLegend, 111703), PE/Cy7 CD11b (BioLegend, 101215), and FITC CD86 (BioLegend, 105005) antibodies. Next, cells were fixed with Fixation Buffer (BioLegend) and permeated with Intracellular Staining Permeabilization Wash Buffer (BioLegend). APC CD206 (BioLegend, 141707) antibody was then used to incubate cells for flow cytometry assay. Macrophages were firstly screened based on the expression of F4/80 and CD11b, and CD86 (M1 marker) and CD206 (M2 marker) expressions were then determined. Data were analyzed by Flowjo Analysis Software.

### Western blot analysis

Total protein was extracted from BMDMs by incubating the cells for 10 min at 4 ℃ in RIPA lysis buffer (Fdbio Science, Guangzhou, China) with additional protease inhibitor. After BCA protein determination (Fdbio Science), 5 × loading buffer (Fdbio Science) was added in protein lysates. Equal quantities of lysates were isolated by SDS-PAGE and transferred onto 0.22 um PVDF microporous membranes (Beyotime Institute of Biotechnology, Jiangsu, China). The membranes were blocked with 5% whole milk and then incubated with primary antibodies for 16 h at 4 °C. Afterwards, the membranes were incubated at room temperature for 60 min with the secondary antibodies. Target protein bands were visualized by FDbio-Dura ECL (Fdbio Science). Antibodies applied for western blot in this study were: rabbit anti-IGF2BP3 (Proteintech, Rosemont, USA, 1:1,000, 14642-1-AP), rabbit anti-iNOS (Proteintech, 1:1,000, 22226-1-AP), rabbit anti-CD206 (Proteintech, 1:1,000, 18704-1-AP), rabbit anti-IL-1β (Proteintech, 1:1,000, 16806-1-AP), mouse anti-IL-4 (Proteintech, 1:1,000, 66142-1-Ig), species-matched HRP-conjugated secondary antibodies (Jackson ImmunoResearch Laboratories, West Grove, PA, USA).

### Immunofluorescence (IF) staining

Mid-sagittal sections (4 μm thick) of paraffin-embedded clinical synovial samples were deparaffinized and rehydrated. Antigen retrieval was conducted by soaking slides in Tris–EDTA pH9.0 in a microwave oven for 10 min. After soaking three times in PBS, slides were administered with 3% hydrogen peroxide for 10 min at room temperature. Next, slides were blocked with 10% bovine serum (Solarbio, Beijing, China) for 1 h at room temperature and incubated with primary antibodies at 4 °C for 16 h. Incubation of fluorescent dye lasted for 1 h at room temperature, then slides were mounted with DAPI mounting medium (Thermo Fisher Scientific). Agents applied for IF assay in this study include: rabbit anti-IGF2BP3 (Proteintech, 1:200, 14642-1-AP), mouse anti-CD68 (Proteintech, 1:200, 66231-2-Ig), species-matched Alexa-488 or -594-labeled secondary antibody (Life Technologies, Carlsbad, CA, USA).

### Cell immunofluorescence staining

BMDMs were seeded onto coverslips in a 12-well plate. After transfected with IGF2BP3 overexpressing plasmids/IGF2BP3 siRNA for 60 h, BMDMs were fixed with 4% paraformaldehyde for 15 min and administered with 0.5% Triton X-100 (Sigma-Aldrich, St. Louis, MO, USA) for 15 min at room temperature. Afterwards, BMDMs were blocked with 10% bovine serum and incubated with primary antibodies at 4 °C for 16 h. After rinsing three times in PBS, coverslips were incubated with fluorescent dye for 1 h at room temperature. Nuclei were stained with DAPI mounting medium. Antibodies used for cell immunofluorescence staining include: rabbit anti-iNOS (Proteintech, 1:150, 22226-1-AP), rabbit anti-CD206 (Proteintech, 1: 150, 18704-1-AP), rabbit anti-IL-1β (Proteintech, 1: 150, 16806-1-AP), mouse anti-IL-4 (Proteintech, 1: 150, 66142-1-Ig), species-matched Alexa-488 or -594-labeled secondary antibody (Life Technologies, Carlsbad, CA, USA).

### Data download

Synovial bulk RNA-seq datasets GSE55235 (including 10 healthy synovium samples and 10 OA synovium samples), GSE55457 (including 10 healthy synovium samples and 10 OA synovium samples), GSE82107 (including 7 healthy synovium samples and 10 OA synovium samples), GSE55584 (including 6 OA synovium samples), and GSE89408 (including 28 healthy synovium samples and 22 OA synovium samples) were downloaded from gene expression omnibus (GEO) database. Synovial scRNA-seq dataset GSE152805 (including 3 OA synovium samples) was obtained from GEO database. RNA-seq data after m6A regulators perturbation was downloaded from RM2target database (http://rm2target.canceromics.org/) [32]. Data of protein interactions was collected from STRING database (https://string-db.org/cgi/input.pl) [33]. Datasets and databases used for each bioinformatic analysis were listed in Table 3.

**Table 3:** Datasets and databases used for bioinformatic analysis

Datasets and databases	Bioinformatic analysis
GSE89408	DEGs analysis
	Differentially m6A regulator expression analysis
	Training set of LASSO-Cox regression modelling
GSE55235, GSE55457	Testing sets of LASSO-Cox regression modelling
GSE89408, RM2target, STRING	PPI network and functional enrichment of m6A regulators and their targeting genes
GSE152805	Dimensionality Reduction and Clustering
	AUCell Scoring of m6A regulator regulated genes in synovial cell clusters
GSE55235, GSE55457, GSE82107, GSE55584, GSE89408	Cell cluster deconvolution
	ssGSEA scoring of m6A regulator regulated genes
	Sample clustering and KEGG functional enrichment based on IGF2BP3 regulated genes

### Differentially expressed genes (DEGs) analysis

The DEGs between normal and OA synovium groups and between C1 and C2 clusters based on IGF2BP3-related genes were identified using the limma package with an FDR-corrected p value < 0.05, fold change (FC) > 1.5 or FC < 1/1.5 [34]. A boxplot of m6A regulators in normal and OA synovium groups was drawn using the function ggboxplot of the R package ggpubr [35]. Volcano plot was drawn with function ggplot of the R package ggplot2 [36]. Heatmap was composed with function pheatmap of the R package pheatmap.

### LASSO (least absolute shrinkage and selection operator)-Cox regression modeling

LASSO-Cox regression modelling was performed using the LASSO-Cox regression tool of Sangerbox, which contained the built-in R package glmnet [37]. The expression matrices of m6A regulators in GSE89408, GSE55235, and GSE5545 were extracted, with m6A regulator expression matrix of GSE89408 as the training set, and m6A regulator expression matrix of GSE55235 and GSE55457 as the testing sets of modelling. The survival times were uniformly set to the same value to cancel the impact of survival on the model since OA is a non-fatal disease, and the status of healthy samples was set as 0, whereas the status of OA samples was set as 1. Receiver operating characteristic (ROC) curve of a single m6A factor was constructed with function roc and plot of the R package pROC and ggplot2 [38].

### Identification of m6A regulator-regulated genes

Potential targeting genes of m6A regulators were identified by downloading RNA-seq data from the RM2target database and setting |logFC|> 2, FDR-corrected p value < 0.05 after m6A regulator perturbation (knock out or knock down). Next, function cor.test of the R package stats was applied to screen co-expression genes whose expression levels were significantly correlated with designated m6A regulators (statistical type set as “spearman” and p value < 0.05). These genes were intersected with RM2target collected genes and sorted according to the absolute value of the correlation coefficient with m6A regulotor expression in descending order to compose the upregulated and downregulated gene sets of each m6A regulator, with a maximum gene number setting of 50.

### Protein–protein interaction (PPI) network construction and hub gene network identification

Key m6A regulators along with their regulated genes were used for PPI network construction using the STRING database (see Additional file 1). Multiple protein mode was selected, the organism was set as “Homo sapiens,” and the minimum interaction score was set at high confidence (0.700). The network visualization tool Cystoscape 3.9.1 software was applied for PPI network topological analysis [39]. Genes were labeled based on their belonging m6A regulators. The cluster query plugin MCODE was used for identifying hub gene network, with the degree cutoff set to 2, node score cutoff set to 0.2, K-core set to 2, and max depth set to 100 [40].

### Hallmark gene set functional enrichment and KEGG enrichment analysis

Genes from the whole and hub gene networks were used for hallmark gene set enrichment analysis. Hallmark gene sets represent 50 specific well-defined biological states or processes and were stored in the Molecular Signatures Database (MSigDB) [41]. Network genes were set as input for enrichment with function enricher of the R package clusterProfiler, with database set as hallmark gene sets, FDR-corrected p value threshold set as 0.05, and were visualized with function GOChord from the R package GOplot [42, 43]. Enrichment analysis of upregulated and downregulated genes of C2/C1 clusters were performed with function enrichKEGG from the R package clusterProfiler, with FDR-corrected p value threshold set as 0.05, and visualized with function dotplot from the R package clusterProfiler.

### scRNA-seq quality check and batch effect removal

The raw gene expression matrices of GSE152805 were converted into a Seurat object using the R package Seurat [44]. Cells with less than 200 expressed genes, over 10,000 expressed genes, or over 20% UMIs derived from the mitochondrial and ribosomal genome were excluded. For the remaining cells, the gene expression matrices were normalized to total cellular read count and to mitochondrial percentage with function NormalizeData, and were standardized with function ScaleData [44].

### Dimensionality reduction and clustering

The function RunPCA was used to calculate the principal components (PCs) [44]. Batch effects were then removed using Harmony [45]. The RunTSNE function in its default setting was applied to visualize the first 20 Harmony-aligned coordinates. Differential gene expression tests were run using the function FindAllMarkers with min.pct set to 0.25 and logfc.threshold set to 0.25. Acknowledged cell markers of synovial cell clusters were collected for cell clustering, and marker genes were visualized with function dotplot [46]. Python package PHATE was then used for denoising reduction [47].

### AUCell scoring of m6A regulator regulated genes in synovial cell clusters

M6A regulators-regulated genes were set as input genes, scored, and visualized with the R package irGSEA. Briefly, function irGSEA.score was used for scoring, with species set as homo sapiens, method set as AUCell, and kcdf set as Gaussian, and then scores were integrated with function irGSEA.integrate. The AUCell score heatmap was created with function irGSEA.heatmap, and the AUCell score feature plots were constructed with function irGSEA.density.scatterplot.

### Merge and batch effect removal of bulk RNA-seq datasets

The GEO datasets GSE55235, GSE55457, GSE82107, GSE55584, and GSE89408 were normalized and set as input data in the batch removal tool of Sangerbox, which contained the built-in R package inSilicoMerging for data merge and the R package sva for batch effect removal, with method set as COMBAT [37, 48].

### Cell cluster deconvolution

The R package MuSiC was applied to determine the cell type proportions in the merged bulk gene expression matrix composing of GSE55235, GSE55457, GSE82107, GSE55584, and GSE89408 [49]. Briefly, function music_prop was used for cell cluster deconvolution, with sc.sce set as the expression matrix of GSE152805.

### ssGSEA scoring of m6A regulator regulated genes

M6A regulator-regulated genes were used as input gene sets. The ssGSEA scores of each sample in the merged bulk-RNA seq dataset were calculated with function gsva of the R package GSVA in its default setting, with method set as ssGSEA [50]. Visualization of ssGSEA scores was performed with function ggboxplot. Person correlation between ssGSEA scores and estimated cell ratio of bulk RNA-seq data was calculated with function corr.test of the R package psych and was visualized with function geom_heat_tri of the R package ggDoubleHeat.

### Sample clustering based on IGF2BP3 regulated genes

The expression matrix of IGF2BP3 upregulated and downregulated genes of the merged bulk RNA-seq dataset was used for sample clustering and visualization with the clustering tool of Sangerbox, which contained the built-in R package ConsensusClusterPlus, with maximum cluster number set to 10, number of subsamples set to 10, proportion of items to sample set to 0.8, and distance set as “pearson” [37, 51]. When k = 2, the cluster-consensus was highest. PCA reduction of C1 and C2 was conducted with function prcomp of the R package stats and visualized with function ggplot2.

### Statistical analyses

Experiments including qPCR, WB, flow cytometry, and cellular fluorescence were conducted in triplicate. The IF results in synovium tissues were estimated by two independent observers. Data are displayed as the mean ± SD. An unpaired Student’s *t*-test was used to compare two groups of data. For data involving more than two groups, a one-way analysis of variance (ANOVA) was performed, followed by Tukey’s post-hoc test. Statistical significance was set as P < 0.05.

## Results

### M6A regulators were dysregulated in OA synovium

**Fig. 1:**
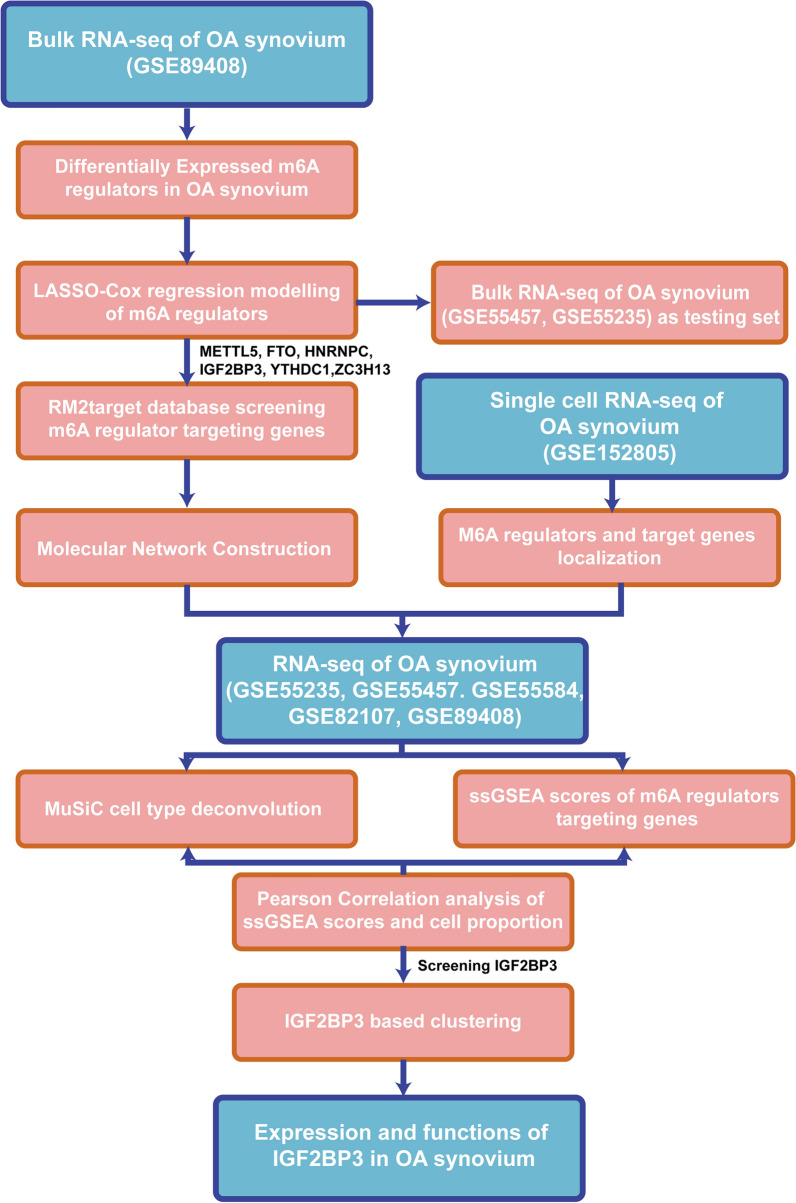
Flow chart of the study. Bulk RNA-seq dataset GSE89408 was downloaded to identify differentially expressed m6A regulators and build LASSO-Cox regression model to screen core m6A regulators in OA synovium. Next, potential targets of these regulators were collected from RM2target database to construct a molecular network indicating their interaction. Afterwards, we used scRNA-seq dataset GSE152805 to identify the localization of core m6A regulators along with their targeting genes. A large merged dataset composed of 5 GEO datasets (GSE55235, GSE55457, GSE82157, GSE55584, and GSE89408) was then used for cell type deconvolution, ssGSEA scoring of m6A regulator targeting genes, and correlation analysis between cell proportion and ssGSEA scores. Above results validated a strong correlation between IGF2BP3 and OA synovial macrophages. Additionally, clustering of samples in merged dataset was performed based on IGF2BP3 targeting genes to speculate differentially enriched pathways between sample clusters. Finally, IGF2BP3 expression was detected in clinical samples, and its functions were explored in BMDMs

This study was conducted according to the flowchart shown (Fig. 1). Briefly, the bulk RNA-seq dataset GSE89408 was downloaded to identify differentially expressed m6A regulators and build a LASSO-Cox regression model to screen core m6A regulators in OA synovium. Next, the potential targets of these regulators were collected from the RM2target database to construct a molecular network indicating their interactions. Next, we used the scRNA-seq dataset GSE152805 to identify the localization of core m6A regulators and their target genes. A merged dataset composed of five GEO datasets (GSE55235, GSE55457, GSE82157, GSE55584, and GSE89408) was used for cell-type deconvolution, ssGSEA scoring of m6A regulator-targeting genes, and correlation analysis between cell proportion and ssGSEA scores. These results validated the strong correlation between IGF2BP3 and OA synovial macrophages. Additionally, samples in the merged dataset were clustered based on the expression of IGF2BP3 targeting genes to identify differentially enriched pathways between high- and low-IGF2BP3 clusters. Finally, IGF2BP3 expression was detected in clinical samples and its functions were explored in BMDMs.

**Fig. 2:**
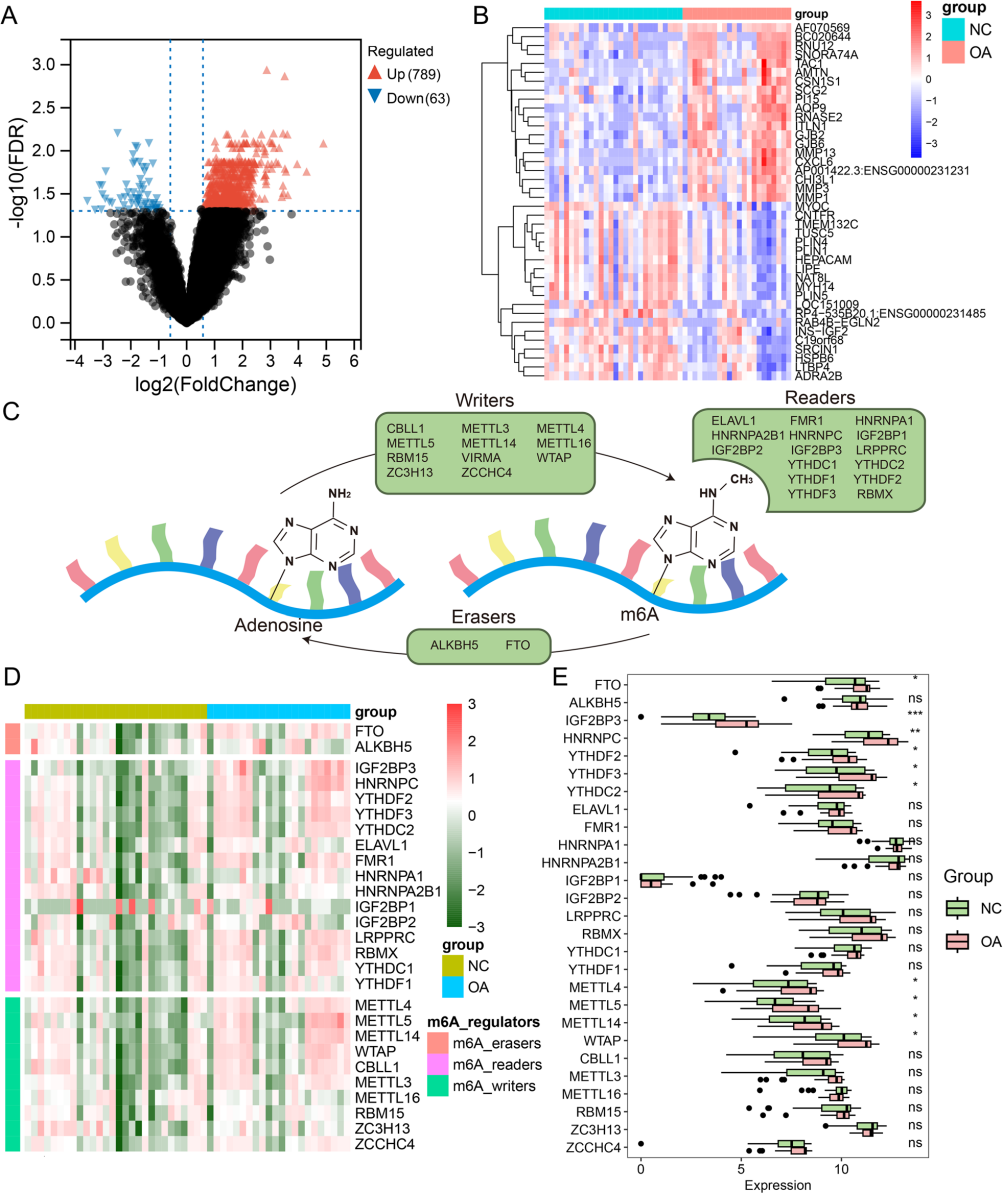
M6A regulators were dysregulated in OA synovium. **A** Volcano plot of differentially expressed genes in OA versus healthy synovium. Red and blue points represent the genes with significantly upregulated or downregulated expressions in OA synovium (FDR < 0.05 and absolute fold-change > 1.5). The two vertical dashed lines denote ± log2(1.5) fold-changes in gene expression, and the horizontal dashed line marks − log10(FDR adjusted p-value cutoff 0.05). **B** Heatmap of top 20 upregulated and downregulated genes in OA synovium. Red and blue elements of sample group bar indicate OA and healthy synovium samples. Red and blue elements in heatmap represent high or low expression of genes. **C** Diagram of m6A modification annotated with key m6A writers, erasers and readers. **D** Heatmap of key m6A regulator expressions in each OA and healthy synovium. Red and green elements in heatmap represent high and low expression of m6A regulators. **E** Boxplot of key m6A regulator expression in OA and control groups. Red and green boxes indicate m6A regulator levels of OA and healthy synovium samples. *P < 0.05, **P < 0.01, ***P < 0.001, ns not significant

First, we aimed to elucidate the alterations in the expression profile and m6A regulator levels in the OA synovium. DEGs analysis of the bulk RNA-seq dataset GSE89408 (containing 28 normal and 22 OA synovial samples) showed 789 upregulated and 63 downregulated genes in OA synovium compared with controls, indicating a distinct expression profile in OA conditions (Fig. 2A). The top 20 enhanced and suppressed genes in the OA synovium were displayed as a heatmap (Fig. 2B). Next, the expression levels of 28 key m6A regulators (including 11 m6A writers, 2 m6A erasers, and 15 m6A readers; Fig. 2C) were determined in individual synovial samples and whole sample groups as heatmaps and boxplots, respectively (Fig. 2D, E). Among these regulators, four writers (METTL4, METTL5, METTL14, and WTAP), one eraser (FTO), and five readers (IGF2BP3, HNRNPC, YTHDF2, YTHDF3, and YTHDC2) were differentially expressed, or more precisely, were all upregulated in the OA synovium, indicating the existence of an overactive m6A regulating process in OA.

### 6 m6A regulators formed an effective prediction model of OA

**Fig. 3:**
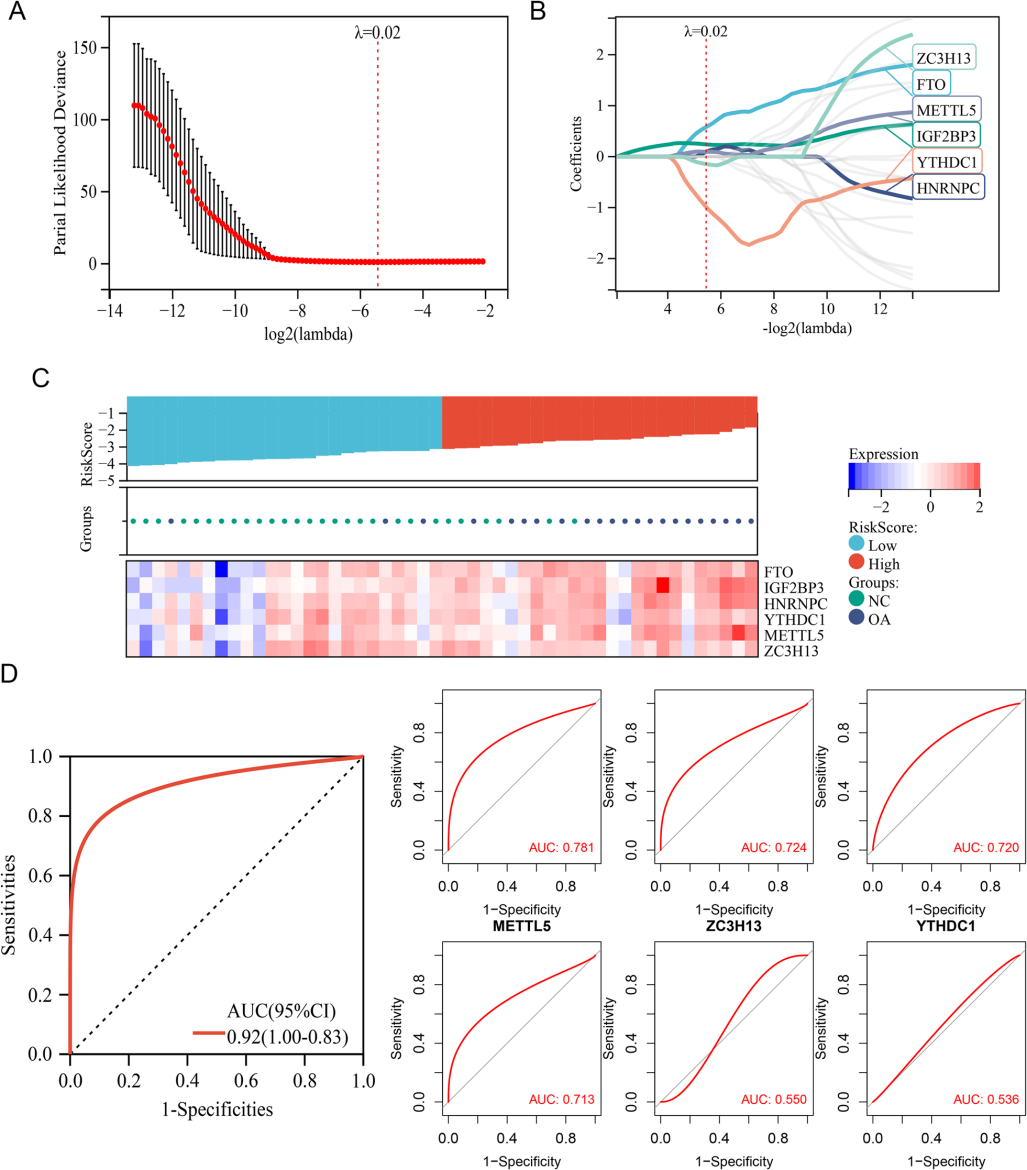
6 m6A regulators formed an effective prediction model of OA. **A** Partial likelihood deviance for LASSO-Cox regression. Lambda value of 0.0230 showed the minimum cross-validation error. **B** The coefficients of model genes were calculated to remove highly correlated genes and 6 m6A regulators (HNRNPC, IGF2BP3, YTHDC1, FTO, METTL5, ZC3H13) were identified by LASSO-Cox regression. **C** Heatmap of OA risk scores and m6A regulator expressions. OA risk scores of samples were sorted in ascending order. Samples whose risk scores ranked top 50% were called high-risk samples (indicated by red bar), while the remaining samples were referred to as low-risk samples (indicated by blue bar). **D** ROC curve for the OA prediction model based on the expression levels of these 6 m6A regulators in training set. **E** ROC curve based on single m6A regulator expression in OA training set

To identify the core m6A regulators associated with OA progression and establish an effective OA prediction model, the GSE89408 dataset was subjected to LASSO-Cox regression analysis, a method for refined predictive modelling and key variable extraction [52]. When the lambda was 0.0230, six regulators (FTO, YTHDC1, METTL5, IGF2BP3, ZC3H13, and HNRNPC) were screened for modelling (Risk Score = 0.582*FTO + 0.093*HNRNPC + 0.240*IGF2BP3 + 0.095*METTL5−0.988*YTHDC1−0.145*ZC3H13, Fig. 3A, B). Figure 3C shows the relationship between the OA risk scores and the expression levels of these six genes. In the training set, the ROC curve showed a large area under the curve (AUC) of 0.92, indicating a high accuracy of OA identification (Fig. 3D). Single-molecule ROC curves of IGF2BP3, HNRNPC, FTO, METTL5, ZC3H13, and YTHDC1 had AUC of 0.781, 0.724, 0.720, 0.713, 0.550, and 0.536, respectively, indicating that factors such as IGF2BP3, HNRNPC, FTO, and METTL5 with AUC > 0.7 showed high diagnostic values alone (Fig. 3D). Next, GSE55235 (comprising 10 control and 10 OA synovium samples) and GSE55457 (comprising 10 control and 10 OA synovium samples) were used as testing sets. As expected, our model performed well to separate control and OA samples, and showed high AUC of 0.88 and 1.00 in testing sets GSE55457 and GSE55235, respectively (Additional file 2: Fig. S1A–D). In summary, a model based on m6A regulators showed high efficacy in predicting OA, and six m6A regulators were identified as key candidate variables closely related to OA. Their expression and functions warrant further investigation.

### The functional network suggested the potential role of core m6A regulators mediating OA synovitis

**Fig. 4:**
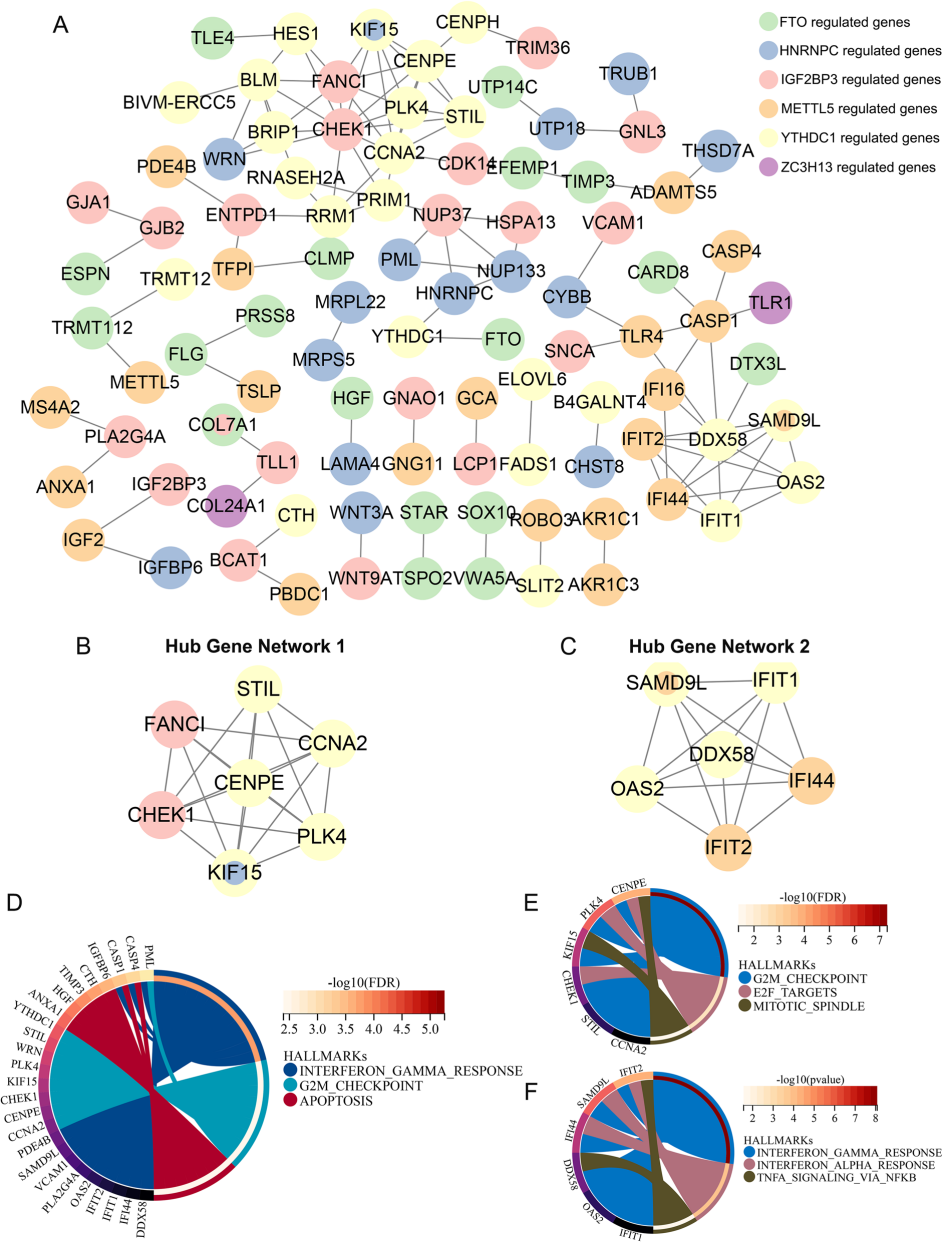
Functional network suggested potential role of core m6A regulators mediating OA synovitis. **A** Molecular network of core m6A regulators and their targeting genes analyzed by STRING database. Nodes in green, blue, pink, orange, yellow, and purple represent FTO-, HNRNPC-, IGF2BP3-, METTL5-, YTHDC1-, ZC3H13-regulated genes. **B**, **C** MCODE application identifying two hub gene networks of the whole molecular network. **D** Hallmark gene set enrichment of the whole molecular network based on 50 specific well-defined biological processes stored in the MSigDB database. Peripheral circle of the chord diagram indicates − log10 (FDR-pValue), and pathways of FDR-pValue < 0.05 were selected. **E**, **F** Hallmark gene set enrichment of the two hub gene networks. Peripheral circle of the chord diagram indicates − log10 (FDR-pValue), and pathways of FDR-pValue < 0.05 were selected

Since one of the major mechanisms of m6A regulators is binding to downstream mRNA and mediating their stability and decay, the possibly targeted mRNAs were further included to build a molecular network based on these six core m6A regulators [16, 17, 53]. Target genes of core m6A regulators in the synovium were screened using the strategy described in the “Methods” section (Additional file 2: Fig. S2A, B), and are displayed in Additional file 2: Fig. S2C (upregulated target genes) and Additional file 2: Fig. S2D (downregulated target genes) (No ZC3H13 downregulated genes were identified). Next, a PPI network was built based on these m6A regulators, along with their potential target genes, to speculate on their interaction and signaling mediation in OA (Fig. 4A). In this network, the FTO-, HNRNPC-, IGF2BP3-, METTL5-, and YTHDC1- regulated genes were extensively interacted at the protein level, whereas few ZC3H13-regulated genes were observed. Subsequently, two hub gene networks were identified using MCODE plugin (Fig. 4B, C). Hub gene network 1 was mainly composed of YTHDC1- and IGF2BP3- regulated genes, whereas hub gene network 2 mainly contained METTL5- and YTHDC1- regulated genes, indicating that these three m6A regulators and their regulated genes were more densely interconnected and may play a more important role in mediating biological processes of synovium. Furthermore, hallmark gene sets representing 50 specific well-defined biological states or processes were used for functional enrichment to identify crucial pathways co-regulated by these core m6A regulators. The 3 significantly enriched pathways of the m6A interaction network were IFN-γ response, G2M checkpoint, and apoptosis, which were highly consistent with the aberrant inflammation and cell cycle in the OA synovium (Fig. 4D). While hub gene network 1 was mainly enriched in cell cycle related pathways (G2M checkpoints, E2M targets, and mitotic spindle), genes of network 2 were strongly correlated with inflammation (IFN-γ, IFN-α, and TNF-α signalling via NF-κB), indicating these 2 hub gene groups can be a functional miniature of the complex m6A network (Fig. 4E, F). In summary, our data show a strong correlation between core m6A regulators and abnormal functions of the OA synovium.

### scRNA-seq revealed the localization of core m6A regulators and their target genes

**Fig. 5:**
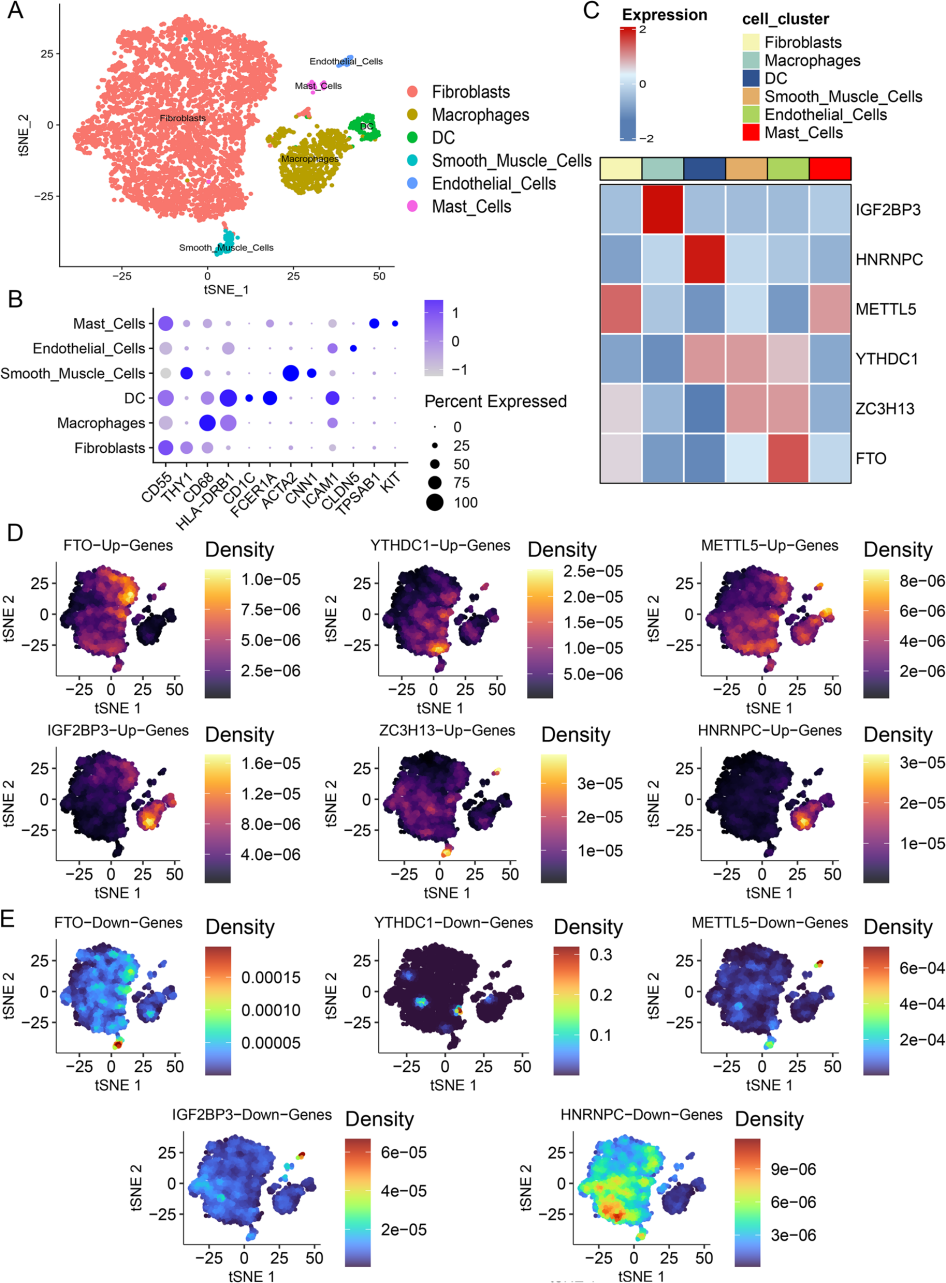
scRNA-seq revealed the localization of core m6A regulators and their targeting genes. **A** t-SNE plot of cells belonging to 3 OA synovium samples, showing six transcriptionally distinct cell clusters. **B** Dot plot of representative marker genes of six synovial cell clusters. **C** Heatmap of six core m6A regulator expressions in synovial cell clusters. **D** Feature plots of AUCell scores of core m6A regulators upregulated genes in each cell cluster. Redder dots represent cells with higher AUCell scores. **E** Feature plots of AUCell scores of core m6A regulators downregulated genes in each cell cluster. Redder dots represent cells with higher AUCell scores

To determine the distribution of these six core m6A factors, public scRNA-seq data GSE152805 of three OA synovial samples were collected and data quality checks and batch effect removal were performed (Additional file 2: Fig. S3A–C). Based on acknowledged cell markers, OA synovial cells were categorized into six cell clusters (Fig. 5A, B). The expression of the six core m6A regulators is displayed in a heatmap and feature plots (Fig. 5C, Additional file 2: Fig. S3D). Here, we mainly focused on the major cell cluster (fibroblasts) and the immunocyte cluster (macrophages) of the synovium because they are the main effector cells in OA synovitis [54]. Relatively high FTO, METTL5, YTHDC1, ZC3H13 levels and a moderate expression of HNRNPC were found in fibroblasts, whereas IGF2BP3 and HNRNPC were highly expressed in macrophages. Subsequently, the AUCell scores of up-/downregulated genes of m6A regulators were evaluated in each cell cluster (Fig. 5D, E, Additional file 2: Fig. S3E). In accordance with IGF2BP3 and HNRNPC distribution, OA synovial macrophages showed high expression of IGF2BP3- and HNRNPC-upregulated genes, whereas IGF2BP3- and HNRNPC-downregulated genes were lowly expressed in OA macrophages, indicating that IGF2BP3 and HNRNPC are potential m6A regulators that mediate macrophages in the OA synovium. Regarding the m6A regulators mainly expressed by fibroblasts, no significant location consistency was found among FTO, METTL5, YTHDC1, and ZC3H13 and their up-/downregulated genes.

### IGF2BP3 was highly correlated with OA synovial macrophages

**Fig. 6:**
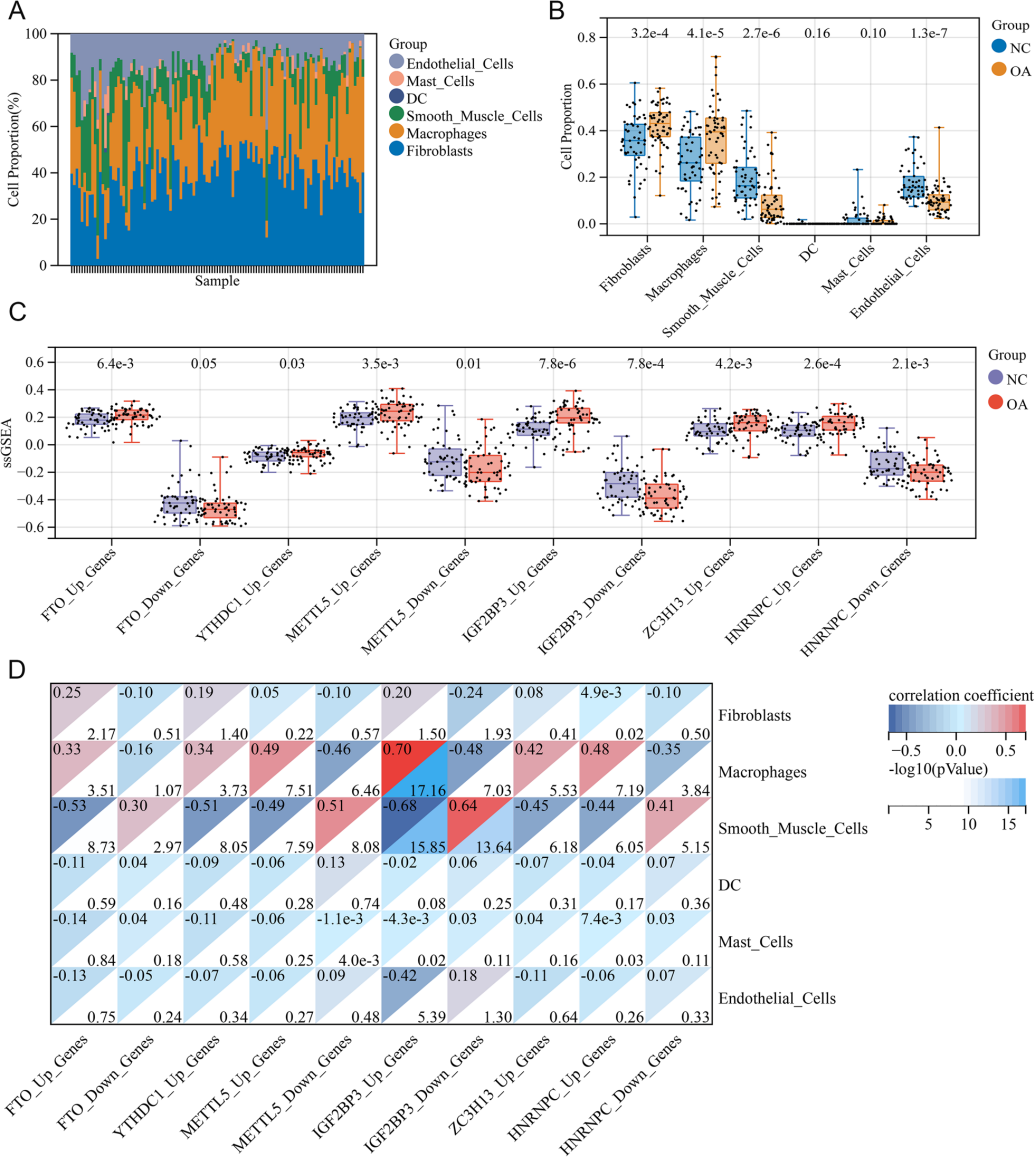
IGF2BP3 was highly correlated with OA synovial macrophages. **A** Cell type proportion in bulk RNA-seq data of 55 normal and 58 OA synovium samples with the MuSiC deconvolution. **B** Box plot of cell proportion in control and OA synovial groups, with numbers at the top representing P value. **C** Boxplot of ssGSEA scores of m6A regulators targeting genes in control and OA synovium, with numbers at the top representing P value. **D** Pearson correlation heatmap of ssGSEA scores of m6A regulators targeting genes and cell clustering. Numbers on the top left of the heatmap blocks represent correlation coefficient, and numbers on the bottom right of the heatmap blocks represent − log10(pValue)

To further validate the role of these m6A regulators in cell clusters of the control/OA synovium, public RNA-seq data of 113 synovium samples (comprising 55 control and 58 OA synovium samples) belonging to the GSE55235, GSE55457, GSE82157, GSE55584, and GSE89408 datasets were collected and merged (Additional file 2: Fig. S4A) and batch effects were removed (Additional file 2: Fig. S4B–E). Next, the MuSiC package was used for bulk cell-type deconvolution based on the scRNA-seq results from GSE152805. The proportion of cells in each synovial sample or whole sample group is displayed in Fig. 6A, B. Compared to the controls, the OA synovium displayed higher ratios of fibroblasts and macrophages, which is a typical manifestation of cell distribution in the OA synovium. Afterwards, we calculated the ssGSEA scores of up-/down-regulated gene sets of the six core m6A factors, and scores of all gene sets were significantly altered in the OA synovium compared to the healthy group (Fig. 6C). Furthermore, Pearson’s correlation test showed the highest (0.70) and lowest (− 0.48) correlation coefficient between ssGSEA scores of IGF2BP3 up-/down-regulated genes and macrophage proportion, respectively, while correlation coefficients of only 0.48 and − 0.35 were observed between ssGSEA scores of HNRNPC up-/down-regulated genes and macrophage proportion, indicating IGF2BP3 was more associated with OA macrophage alterations than HNRNPC (Fig. 6D). For fibroblasts, no strong correlation was found between the ssGSEA score of any gene set and fibroblast distribution. Taken together, our analysis further demonstrates that IGF2BP3 may play an important role in regulating macrophages in the OA synovium.

### IGF2BP3 was associated with OA synovial phenotypic changes

**Fig. 7:**
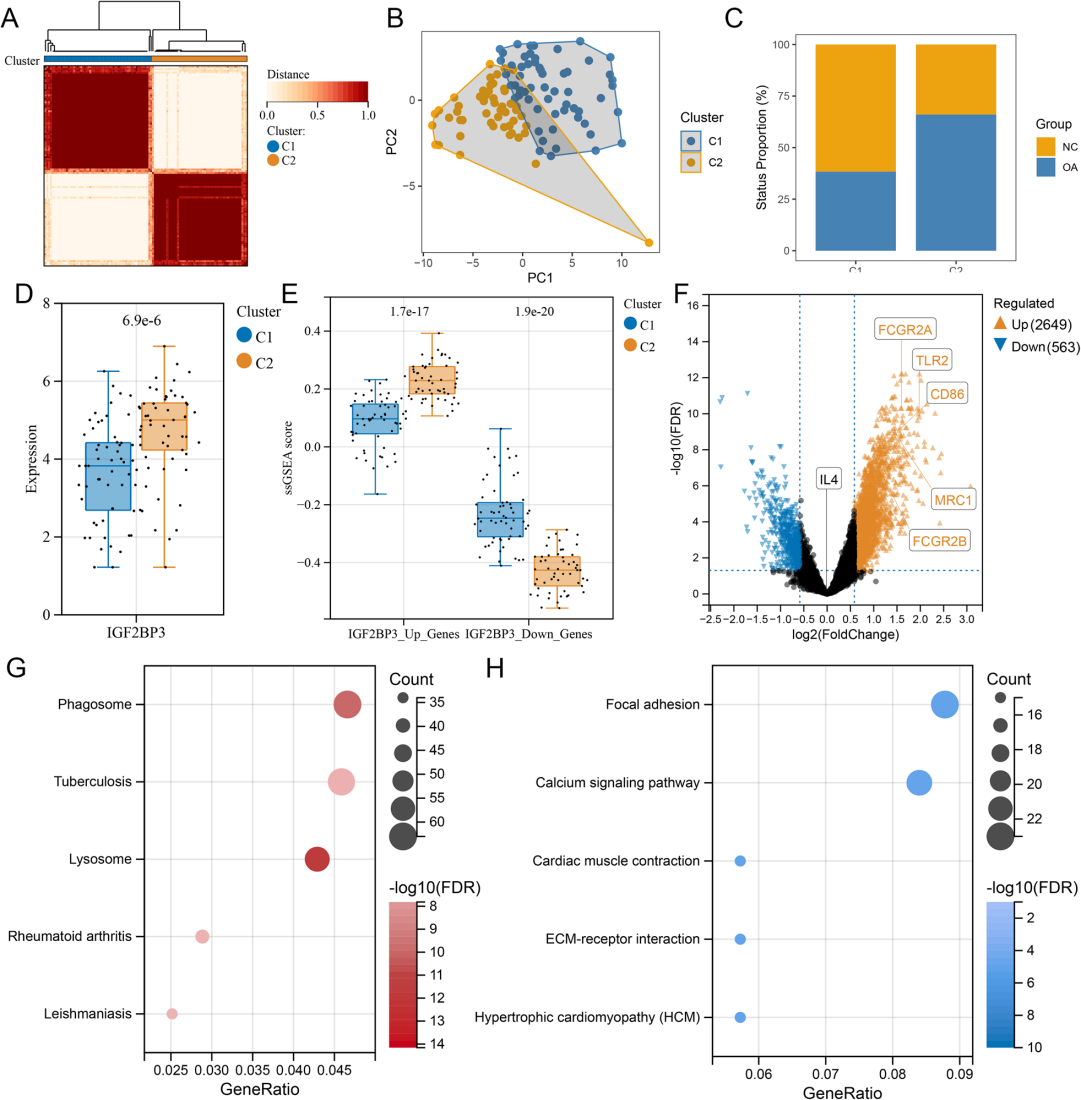
IGF2BP3 was associated with OA synovial phenotypic changes. **A** Heatmap of two synovium sample clusters identified with function ConsensusClusterPlus based on IGF2BP3 and its targeting genes, C1 cluster contains 60 synovium samples and C2 cluster contains 53 synovium samples. **B** PCA reduction of C1 and C2 clusters. **C** Proportion of control and OA samples in C1 and C2 clusters. **D** IGF2BP3 expression in C1 and C2 clusters, with numbers on the top indicating P value. **E** ssGSEA scores of IGF2BP3 upreuglated/downregulated genes in C1 and C2 clusters, with number on the top indicating P value. **F** Volcano plot of DEGs in C1 and C2 clusters marked with macrophage (FCGR1A, FCGR1B), M1 macrophage (CD86, TLR2), and M2 macrophage (MRC1, IL-4) markers. Brown and blue points represent the genes with significantly upregulated or downregulated expressions in C2 synovium compared to C1 (FDR < 0.05 and absolute fold-change > 1.5). The two vertical dashed lines denote ± log2(1.5) fold-changes in gene expression, and the horizontal dashed line marks − log10(FDR adjusted p-value cutoff 0.05). **G** Top 5 enriched KEGG pathways of upregulated genes in C2 cluster compared to C1 cluster. **H** Top 5 enriched KEGG pathways of downregulated genes in C2 cluster compared to C1 cluster

Subsequently, we clustered the 113 synovial samples based on the expression level of IGF2BP3 along with its up-/downregulated genes. Based on the highest consensus values, the samples were divided into two clusters [C1(N = 60) and C2(N = 53)] (Additional file 2: Fig. S5A, Fig. 7A). PCA revealed an approximate separation of the two clusters, indicating distinct gene expression patterns (Fig. 7B). Among these, 23 (39.7%) and 35 (60.3%) samples belonging to the C1 and C2 clusters, respectively, were OA synovium, indicating that the C2 cluster was more likely to possess OA features than C1 (Fig. 7C). Moreover, higher IGF2BP3 expression and higher/lower ssGSEA scores of IGF2BP3 up/downregulated genes were detected in the C2 cluster (Fig. 7D, E). Next, we compared the DEGs in the C1 and C2 clusters. As shown in Fig. 7F, the C2 cluster showed a higher expression of markers of primitive M0 macrophages (FGCR1A and FGCR1B) and proinflammatory M1 macrophages (CD86 and TLR2). However, marker of anti-inflammatory M2 macrophage (MRC1) was also increased in the C2 cluster, although to a lesser extent than M1 markers, whereas the anti-inflammatory factor IL-4 did not show a significant change. To speculate IGF2BP3-associated pathways in the OA synovium, KEGG pathway enrichment analysis was performed on the DEGs between the C1 and C2 clusters. Enhanced genes in the C2 cluster were mainly enriched in macrophage-regulated pathways and macrophage-involved diseases, such as phagosome and lysosome pathways, RA, tuberculosis, and leishmaniasis (Fig. 7G). The suppressed genes in C2 were mainly enriched in ECM-related pathways such as focal adhesion and ECM-receptor interaction pathways (Fig. 7H). In summary, the RNA modification process mediated by IGF2BP3 showed a significant correlation with OA macrophage phenotypic changes.

### IGF2BP3 was upregulated in OA synovium

**Fig. 8:**
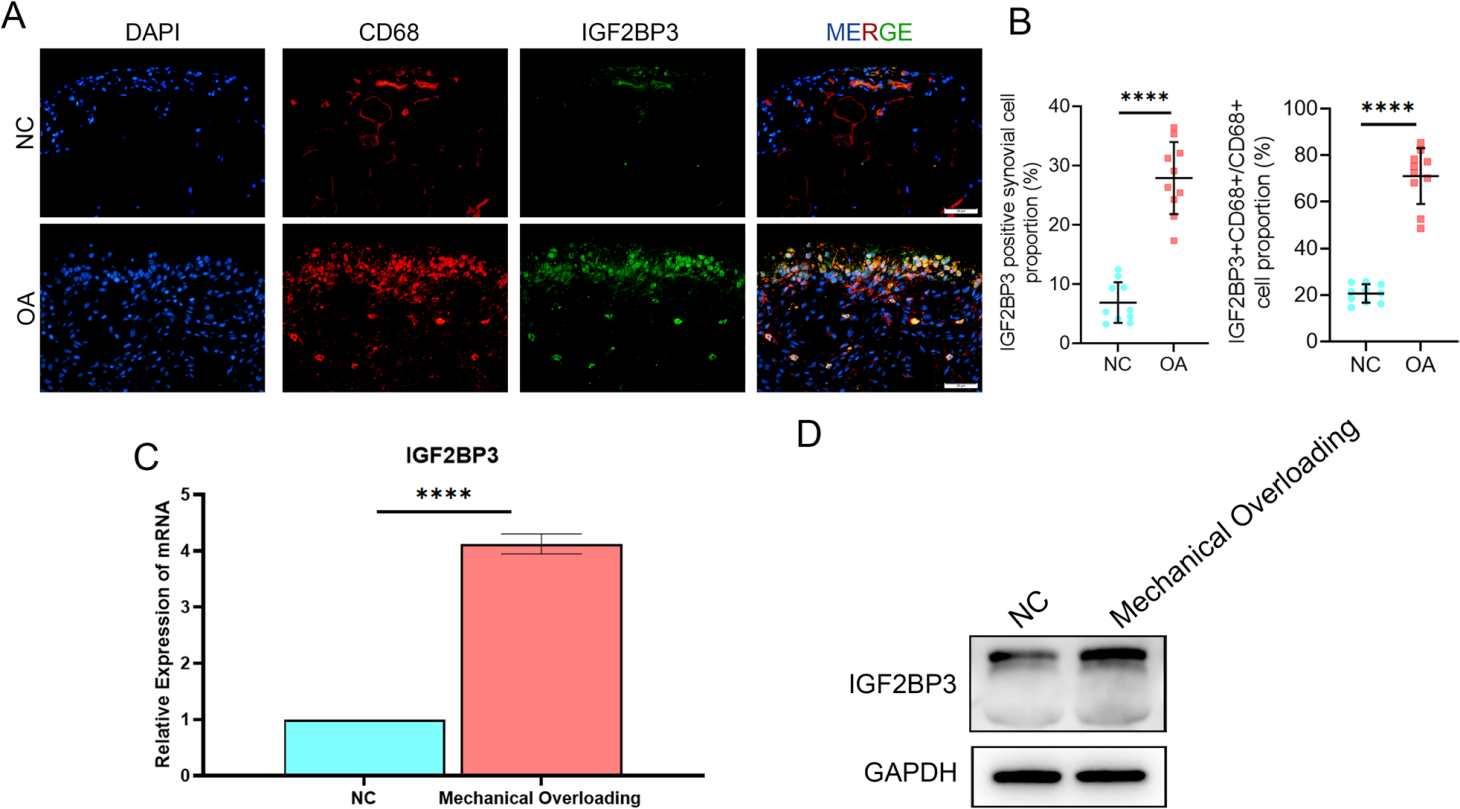
IGF2BP3 was upregulated in OA synovium. **A**, **B** Immunofluorescence (**A**) and quantification (**B**) of IGF2BP3 in control and OA clinical synovial samples, n = 10 per group, Scale bars: 25 μm. **C** qPCR assay of IGF2BP3 in mechanical overloading BMDMs (n = 3). **D** Immunoblotting of IGF2BP3 in mechanical overloading BMDMs (n = 3). ****P < 0.0001

To validate the expression level and localization of IGF2BP3 in the synovium, clinical synovial specimens of OA were collected (n = 10), with synovial samples from patients during arthroscopy for trauma or joint derangements as controls (n = 10); no significant differences in sex, age, and BMI were observed between the two groups (Table 1). High IGF2BP3 expression was detected in OA samples, as predicted, and IGF2BP3 strongly colocalized with CD68, a macrophage marker (Fig. 8A, B). To our surprise, in vitro assay showed IL-1β or LPS stimulated BMDMs did not show strong elevation of IGF2BP3 (Additional file 2: Fig. S6A, B), whereas IGF2BP3 was significantly upregulated in macrophages after mechanical overloading (Fig. 8C, D). In summary, IGF2BP3 levels are enhanced in clinical OA synovial samples, and mechanical factors, rather than inflammatory factors, may be the main factors promoting its expression.

### IGF2BP3 promoted M1 macrophage polarization in OA

**Fig. 9:**
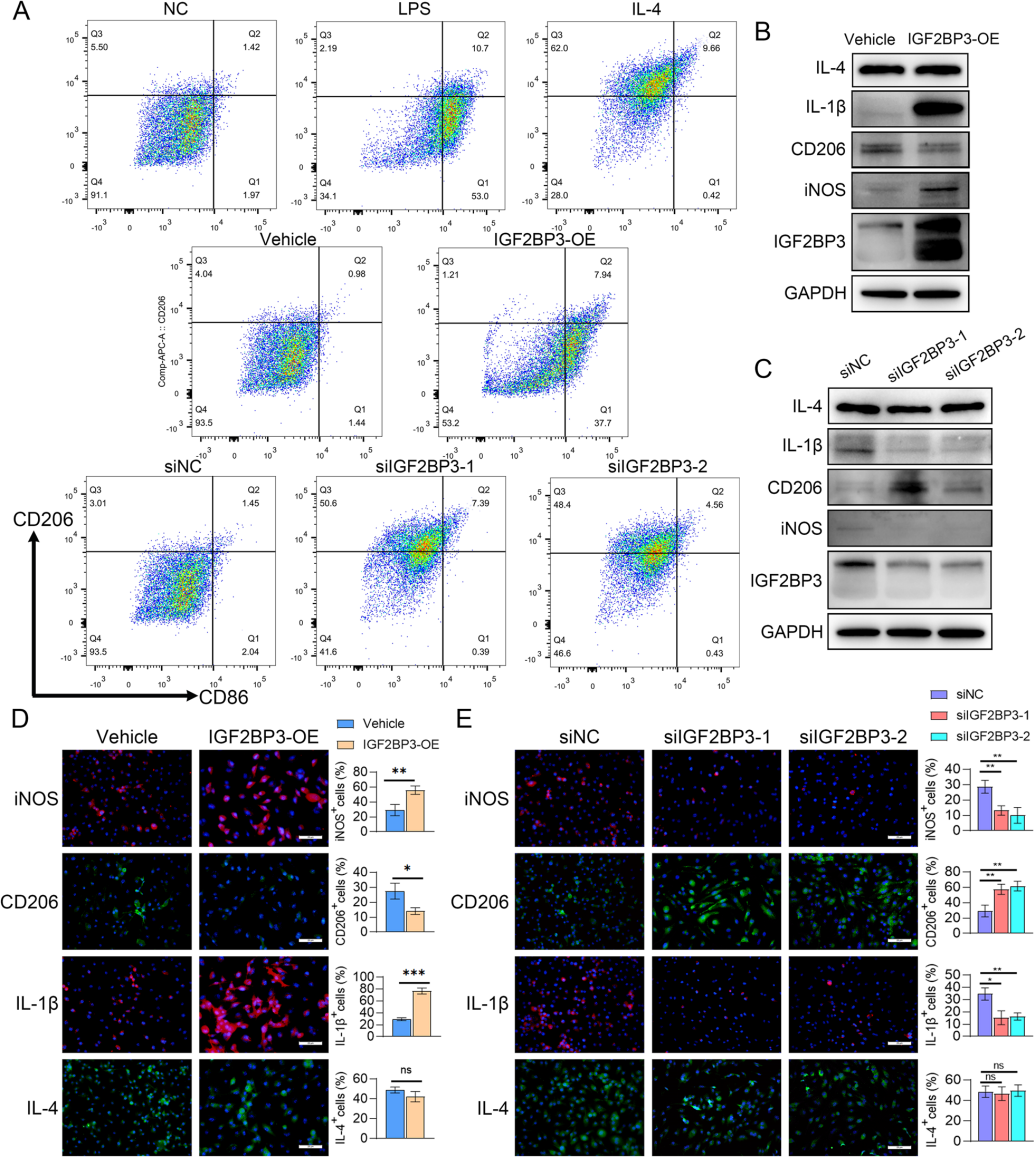
IGF2BP3 promoted M1 macrophage polarization in OA. **A** Flow cytometry analysis of CD206 and CD86 expression of BMDMs after LPS treatment (positive control of M1), IL-4 treatment (positive control of M2), IGF2BP3 overexpression, and IGF2BP3 knock down (n = 3). **B** Immunoblotting of IGF2BP3 expression, polarization (iNOS, CD206), inflammation (IL-1β) and anti-inflammation (IL-4) genes in IGF2BP3 overexpressed BMDMs (n = 3). **C** Immunoblotting of IGF2BP3 expression, polarization (iNOS, CD206), inflammation (IL-1β) and anti-inflammation (IL-4) genes in IGF2BP3 knock down BMDMs (n = 3). **D** Cell fluorescence of polarization (iNOS, CD206), inflammation (IL-1β) and anti-inflammation (IL-4) genes in IGF2BP3 overexpressed BMDMs (n = 3), Scale bars: 25 μm. **E** Cell fluorescence of polarization (iNOS, CD206), inflammation (IL-1β) and anti-inflammation (IL-4) genes in IGF2BP3 knock down BMDMs (n = 3), Scale bars: 25 μm. *P < 0.05, **P < 0.01, ***P < 0.001, ****P < 0.0001, ns not significant

Finally, the levels of M1 macrophage markers (CD86 and iNOS) and proinflammatory genes (IL-1β, IL-6, TNF-α), as well as M2 macrophage marker (CD206) and anti-inflammatory genes (IL-4, IL-10) were detected after IGF2BP3 intervention to identify its regulatory effects on M1 or M2 phenotypes in BMDMs. qPCR assay showed higher expressions of the murine M1 macrophage marker (iNOS) and inflammatory factors (IL-1β, IL-6) and lower levels of M2 macrophage marker (CD206) after IGF2BP3 overexpression in BMDMs (Additional file 2: Fig. S7A). In contrast, after feasible siRNAs targeting IGF2BP3 were selected, BMDMs with IGF2BP3 knockdown tended to polarize in the M2 subtype rather than M1 and showed less transcription of inflammatory cytokines (Additional file 2: Fig. S7B). However, the anti-inflammatory factors (IL-4 and IL-10) did not show obvious alterations upon IGF2BP3 overexpression or inhibition in BMDMs. Flow cytometry analysis revealed that the proportion of CD206^−^CD86^+^ (M1) macrophages remarkably increased after IGF2BP3 overexpression, while a higher proportion of CD206^+^CD86^−^ (M2) macrophages was found in IGF2BP3 knockdown BMDMs (Fig. 9A). Accordingly, WB assay showed higher expression of M1 markers and proinflammatory genes, along with lower M2 marker levels in IGF2BP3 overexpressed BMDMs, while IGF2BP3 silencing inhibited the expression of M1 markers and inflammatory genes, and enhanced the expression of M2 markers at the protein level (Fig. 9B, C). We also performed cellular fluorescence staining to further validate the phenotypes induced by IGF2BP3 interventions (Fig. 9D, E). In conclusion, these results suggest that IGF2BP3 plays an essential role in promoting M1 macrophage polarization and inflammation.

## Discussion

In this study, we determined the expression pattern of key m6A regulators in the OA synovium, and six of these m6A regulators (FTO, YTHDC1, METTL5, IGF2BP3, ZC3H13, and HNRNPC) were used to build a high-fitting OA prediction model. Based on these six factors and their potential mRNA targets, we constructed a molecular network and found that they are involved in inflammation and cell cycle regulation in the OA synovium. In combination with the scRNA-seq data, we determined that IGF2BP3 expression was most strongly correlated with phenotypic alterations in macrophages. Further studies verified the increased IGF2BP3 expression in OA synovial macrophages and demonstrated that IGF2BP3 promoted synovial inflammation and M1 macrophage polarization, which identified a pivotal m6A regulator modulating OA progression.

Synovitis is an important pathological condition highly correlated with OA onset and progression. As a sensible symptom occurring in the very early stage of OA, synovial inflammation can be found even before detectable cartilage damage [55]. Clinical research has shown that a greater effusion-synovitis volume was found 2 years before disease onset in over 50% of patients and was deemed a high-risk factor for accelerated OA [56]. In addition, a study that included 104 clinical subjects proved that synovitis is also a characteristic of late-stage OA [57]. More importantly, the presence of synovitis indicates a nine-fold greater risk of painful knee OA and a faster process of cartilage destruction [58]. Hence, synovitis scoring is a potential tool for predicting and evaluating OA, and synovitis clearance may be a promising method for OA treatment. Based on public RNA-seq data, we found 789 upregulated and 63 downregulated genes in the OA synovium compared to the controls, indicating that the OA synovium had markedly different gene expression patterns. At the cellular level, scRNA-seq data analysis indicated that fibroblasts and macrophages accounted for most OA synovial cells, while smooth muscle cells, endothelial cells, mast cells, and DCs also existed but to a lesser extent. Using the deconvolution algorithm, we validated that fibroblasts and macrophages were the two main cell clusters with enhanced cell proportions in the OA synovium compared to control samples. As the major OA synovial immune cells, macrophages exhibit abnormalities not only in cell number but also in activation status. Activated macrophages can be classified into classically activated M1 macrophages or alternatively activated M2 macrophages, which differ in their responses to microenvironmental stimuli. M1 macrophages are characterized by CD86 (or iNOS in mice) marker genes and enhanced production of proinflammatory cytokines, such as TNF-α, IL-1, IL-6, and IL-12 [59]. M2 macrophages, also known as wound-healing macrophages, are marked by the CD206 gene and display anti-inflammatory secretion phenotypes such as IL-4 and IL-10 [60]. In OA clinical synovial samples, a more than two-fold increase in M1 macrophages was found with a concomitant decrease in M2 macrophages, and this imbalance of M1/M2 polarization was proven to positively modulate synovial inflammation, cartilage destruction, osteophyte formation, and ultimately OA progression [61, 62]. Drugs that mediate macrophage polarization have shown promising therapeutic effects in patients with OA. Transient receptor potential vanilloid 1 (TRPV1) activation is closely related to the alleviation of the pain sensation and inhibition of M1 macrophage polarization [63]. Intra-articular injection of the TRPV1 agonist CNTX-4975 was studied in a phase IIb clinical trial in OA patients and displayed the effects of reduction of pain scores [63]. TissueGene-C promotes the shift of synovial M2 macrophages in the joints and enhances their anti-inflammatory activity, ultimately reducing pain and promoting cartilage regeneration, and a phase III trial of this therapy in OA patients is ongoing [64]. However, current drugs can only partially inhibit inflammatory macrophage formation and OA development, and the molecular mechanisms regulating OA macrophage polarization require further studies to determine more effective and precise therapeutic targets.

M6A modification is the most common posttranscriptional modification in mammals [65]. They participate in modulating biological processes such as mRNA splicing, localization, translation, and stability adjustment [66]. Aberrant m6A modifications hamper gene expression and cell function and ultimately cause diseases, including OA [16]. However, the mode of m6A regulator expression in OA synovium remains unclear. By analyzing public RNA-seq data of OA and normal synovial samples, we identified 10 out of 28 m6A regulators that were upregulated in the OA synovium, indicating a relatively active m6A modification process in OA. Among the 28 m6A regulators, 6 (FTO, YTHDC1, METTL5, IGF2BP3, ZC3H13, and HNRNPC) comprised a well-fitting regression model showing high OA predictive efficiency in both the training and testing datasets, indicating that they may be promising OA biomarkers and effectors. PPI network analysis showed that these m6A regulator-regulated genes interacted extensively at the protein level, while YTHDC1-, IGF2BP3-, and METTL5-regulated genes were identified as hub genes, indicating they owned larger number of interactions and may play a more crucial role in phenotypic regulation in synovium. In addition, the nodes of these PPI networks were highly enriched in pathways contributing to OA synovitis, mainly inflammation pathways (IFNγ, IFNα, and TNF pathways), as well as proliferation and cell cycle-related pathways (G2M checkpoint, apoptosis, mitotic spindle, E2F targets pathways) [67, 68]. Previous studies have identified HNRNPC, FTO, and YTHDC1 as important m6A regulators mediating IFN and TNF secretion and responses, whereas all six m6A regulators have been reported to be closely associated with proliferation and cell cycle regulation in other diseases, which further confirmed our results [69–72]. To determine the localization of these six regulators and their target genes, scRNA-seq dataset was used for joint analysis. No strong correlation of cellular localization in fibroblasts was found among the upregulated genes, downregulated genes, and their corresponding m6A regulator expression. While in synovial macrophages, both IGF2BP3 and IGF2BP3 upregulated genes were highly expressed, whereas IGF2BP3 suppressed genes showed low expression profiles, indicating that IGF2BP3 may serve as a specific m6A regulator in synovial macrophages.

Previous studies have found that m6A modulation is strongly correlated with macrophage aberrance and has great potential as a drug target for modulating macrophage phenotypes. For example, m6A reader YTHDF2 deactivates MAP2K4 and MAP4K4 to inhibit MAPK and NF-κB signaling thus alleviating inflammation in macrophages [73]. The m6A writer METTL3 induces M1 polarization while suppressing M2 polarization, and its highly selective inhibitor STC-15 is approaching phase I clinical trials for cancer [74, 75]. For the m6A reader IGF2BP3, we found that the ssGSEA score of IGF2BP3 upregulated genes was markedly higher, whereas the score of IGF2BP3 downregulated genes was lower in the OA synovium than in controls. Both scores significantly correlated with the proportion of macrophages. Subsequently, synovial samples were categorized into two clusters based on expressions of IGF2BP3 and its target genes: Cluster C2 showed a higher proportion of OA samples, a higher expression of IGF2BP3, a higher ssGSEA score of IGF2BP3-upregulated genes, and a lower score of IGF2BP3-downregulated genes compared to C1. Interestingly, cluster C2 expressed more macrophage markers and displayed a higher ratio of M1 marker gene/M2 marker gene. Moreover, Enhanced genes in C2 were enriched in macrophage-regulated pathways and macrophage-involved diseases, such as phagosome and lysosome pathways, RA, tuberculosis, and leishmaniasis [76–78]. The suppressed genes in C2 were mainly enriched in ECM-related pathways, such as focal adhesion and ECM-receptor interaction pathways, indicating a lack of repair and remodeling of the ECM (a function of M2-polarized macrophages) [79]. To validate our results, we examined the IGF2BP3 expression under OA conditions. As expected, IGF2BP3 expression was markedly upregulated in OA synovial macrophages in human synovial samples. However, LPS and IL-1β, the inflammation stimulators, could not induce IGF2BP3 increase in BMDMs, while mechanical overloading strongly enhanced IGF2BP3 level, indicating mechanical factor may be the main trigger promoting IGF2BP3 in OA macrophages. Several studies have investigated the role of IGF2BP3 in modulating macrophage phenotype, while the results have been conflicting. Some researchers claimed that IGF2BP3 promotes proinflammatory M1 macrophage polarization, while others deemed it to enhance M2 subtype shifting [80, 81]. In our study, we found IGF2BP3 overexpressed BMDMs polarized into the M1 phenotype and secreted more inflammatory cytokines, whereas IGF2BP3 knockdown led to M2 polarization and inhibited inflammation. Nevertheless, neither the overexpression nor silencing of IGF2BP3 changed the expression levels of anti-inflammatory mediators. The possible reasons for these different conclusions may be the use of different cell models or different levels of gene overexpression and knockdown. This suggests that IGF2BP3 may serve as a fine-tuning regulator of macrophage polarization and warrants further investigation. Furthermore, given that mechanical overload induces IGF2BP3 expression, it may play a crucial role as a hub gene in converting mechanical stimuli into inflammatory signals, indicating its potential as a therapeutic target in the OA synovium.

Several studies have also explored the expression patterns of m6A regulators in arthritis via bioinformatics analysis. Ni et al. analyzed the expression levels of 23 m6A regulators in OA chondrocytes and identified YTHDF3 and IGF2BP3 as upregulated m6A readers in OA chondrocytes and may be correlated with enhanced chondrocyte ECM catabolism [30]. Zhao et al. found that IGF2BP3 is enhanced in the synovium of patients with RA and is a potential regulator of inflammation-related pathways [80]. Xiong et al. identified 12 differentially expressed m6A genes in the OA synovium based on RNA-seq data from 10 normal and 10 OA samples, including IGF2BP3 [82]. Combining these studies with our results, it is evident that m6A modulation and IGF2BP3 expression were dysregulated in both OA and RA conditions and affected functions not only in the synovium but also in the cartilage. However, compared to previous studies, our research has unique strengths and novelty. First, we collected as much public RNA-seq data from healthy and OA synovial samples as possible, including 55 normal and 57 OA synovial samples, to make our bioinformatics analysis more convincing. Second, we conducted a novel joint analysis of bulk RNA-seq and scRNA-seq to identify the m6A regulatory pattern of each synovial cell cluster in OA for the first time and identified IGF2BP3 as specifically expressed and functioning factor in synovial macrophages. Third, we included potential mRNA targets of m6A regulators from the RM2target database to create a more comprehensive functional network. Finally, we conducted experiments to verify the clinical correlation between IGF2BP3 levels and OA, and to clarify its role in regulating synovial macrophage polarization. Nevertheless, our study still has some limitations. First, the lack of scRNA-seq data for the normal synovium may introduce deviations when deconvoluting normal synovium samples. Second, the potential targets of m6A regulators were collected from the RM2target database comprising data from other human cell lines. Thus, the interaction between m6A regulators and their predicted binding mRNA in macrophages should be experimentally verified. Third, IGF2BP3 knockout transgenic mice were not included in our current study, which would be supplemented in future to further clarify our conclusions.

## Conclusions

In conclusion, our research identified the expression patterns of m6A regulators and depicted a molecular network based on core m6A genes in the OA synovium. Moreover, we revealed that the m6A reader IGF2BP3 partially functions in OA synovial macrophages by promoting M1 polarization and inflammation. Our study sheds light on the roles of m6A regulators in the OA synovium and preliminarily indicates how IGF2BP3 modulates OA macrophages, thus providing new targets for OA diagnosis and treatment.

## Supplementary Information

**Additional file 1. **m6A_regulator_regulated_genes, STRING_results, Network_nodes. This additional file contains upregulated and downregulated genes of 6 core m6A regulators, PPI result of these genes based on STRING database, and nodes of whole and hub PPI networks which were used for functional enrichment.

**Additional file 2: Figure S1.** (A) OA probabilities (Prob-min) of Lasso-Cox model in testing set GSE55457. (B) ROC curve for the OA prediction model based on the expression levels of 6 m6A regulators in testing set GSE55457. (C) OA probabilities (Prob-min) of Lasso-Cox model in testing set GSE55235. (D) ROC curve for the OA prediction model based on the expression levels of 6 m6A regulators in testing set GSE55235. **Figure S2.** (A, B) Screening strategy of m6A up/down genes. (C) Illustration of METTL5-, FTO-, YTHDC1-, IGF2BP3-, HNRNPC-, and ZC3H13- upregulated genes. (D) Illustration of METTL5-, FTO-, YTHDC1-, IGF2BP3-, and HNRNPC- downregulated genes. Nodes in green, blue, pink, orange, yellow, and purple represent FTO-, HNRNPC-, IGF2BP3-, METTL5-, YTHDC1-, ZC3H13-regulated genes. **Figure S3. **(A) Correlation between gene expression levels (nCount_RNA) and ribosome gene proportion (percent.rb), and correlation between gene expression levels (nCount_RNA) and gene numbers (nFeature_RNA) of synovium samples of of scRNA-seq dataset GSE152805. (B) t-SNE plot of synovial cells in each sample after batch effects removal with Harmony. (C) Gene numbers (nFeature_RNA), gene expression levels (nCount_RNA), mitochondria gene proportion (percent.mt), and ribosome gene proportion (percent.rb) in each synovium sample. Cells with either fewer than 200 expressed genes, more than 10,000 expressed genes, or over 20% UMIs derived from mitochondrial and ribosomal genome were excluded. (D)t-SNE plots of METTL5, FTO, IGF2BP3, HNRNPC, YTHDC1, and ZC3H13 expressions. (E) Heatmap of AUCell scores of core m6A regulators upregulated and downregulated genes in each cell cluster. **Figure S4. **(A) Sample numbers of control and OA synovium in each public bulk RNA-seq dataset. (B) Gene expression levels of each synovial sample before batch effect removal. (C) Gene expression levels of each synovial sample after batch effect removal. (D) UMAP plot of synovial samples before batch effect removal. (E) UMAP plot of synovial samples after batch effect removal. **Figure S5. **(A) Consensus values of different clustering numbers of synovial samples. When k=2, averaged cluster-consensus reached the highest. **Figure S6. (**A) qPCR analysis of IGF2BP3 in LPS treated BMDMs (n=3). (B) qPCR analysis of IGF2BP3 in IL-1β treated BMDMs (n=3). ns not significant. **Figure S7. **(A) qPCR assay of IGF2BP3 expression, polarization (iNOS, CD206), inflammation (IL-1β, IL-6, TNF) and anti-inflammation (IL-4, IL-10) genes in IGF2BP3 overexpressed BMDMs (n=3). (B) qPCR assay of IGF2BP3 expression, polarization (iNOS, CD206), inflammation (IL-1β, IL-6, TNF) and anti-inflammation (IL-4, IL-10) genes in IGF2BP3 knock down BMDMs (n=3). * P < 0.05, **P < 0.01, ****P < 0.0001, ns not significant.

## Data Availability

All data generated or analyzed during this study are included in this published article and its supplementary information files. Scripts and data files for this study are available at https://doi.org/10.5281/zenodo.7866614.

## Abbreviations

IGF2BP3: Insulin-like growth factor 2 mRNA-binding protein 3
OA: Osteoarthritis
RA: Rheumatoid arthritis
m6A: N6 methyl adenosine
messenger RNA: MRNA
scRNA-seq: Single-cell RNA sequencing
RNA-seq: RNA sequencing
BMI: Body mass index
FC: Fold change
M-CSF: Macrophage colony stimulating factor
ROC: Receiver operating characteristic
AUC: Area under the curve
MSigDB: Molecular Signatures Database
PPI network: Protein–protein interaction network
DEG: Differentially expressed gene
IL-1β: Interleukin-1β
LPS: Lipopolysaccharide
BMDM: Bone marrow derived macrophage
ECM: Extracellular matrix
