# Supplementary material for: Expression pattern analysis of m6A regulators reveals IGF2BP3 as a key modulator in osteoarthritis synovial macrophages
Source: J Transl Med. 2023 May 22;21:339. doi: 10.1186/s12967-023-04173-9 (PMC10204300; doi:10.1186/s12967-023-04173-9)
Supplement: Supplementary file 2 — Additional file 2: Figure S1. (A) OA probabilities (Prob-min) of Lasso-Cox model in testing set GSE55457. (B) ROC curve for the OA prediction model based on the expression levels of 6 m6A regulators in testing set GSE55457. (C) OA probabilities (Prob-min) of Lasso-Cox model in testing set GSE55235. (D) ROC curve for the OA prediction model based on the expression levels of 6 m6A regulators in testing set GSE55235. Figure S2. (A, B) Screening strategy of m6A up/down genes. (C) Illustration of METTL5-, FTO-, YTHDC1-, IGF2BP3-, HNRNPC-, and ZC3H13- upregulated genes. (D) Illustration of METTL5-, FTO-, YTHDC1-, IGF2BP3-, and HNRNPC- downregulated genes. Nodes in green, blue, pink, orange, yellow, and purple represent FTO-, HNRNPC-, IGF2BP3-, METTL5-, YTHDC1-, ZC3H13-regulated genes. Figure S3. (A) Correlation between gene expression levels (nCount_RNA) and ribosome gene proportion (percent.rb), and correlation between gene expression levels (nCount_RNA) and gene numbers (nFeature_RNA) of synovium samples of of scRNA-seq dataset GSE152805. (B) t-SNE plot of synovial cells in each sample after batch effects removal with Harmony. (C) Gene numbers (nFeature_RNA), gene expression levels (nCount_RNA), mitochondria gene proportion (percent.mt), and ribosome gene proportion (percent.rb) in each synovium sample. Cells with either fewer than 200 expressed genes, more than 10,000 expressed genes, or over 20% UMIs derived from mitochondrial and ribosomal genome were excluded. (D)t-SNE plots of METTL5, FTO, IGF2BP3, HNRNPC, YTHDC1, and ZC3H13 expressions. (E) Heatmap of AUCell scores of core m6A regulators upregulated and downregulated genes in each cell cluster. Figure S4. (A) Sample numbers of control and OA synovium in each public bulk RNA-seq dataset. (B) Gene expression levels of each synovial sample before batch effect removal. (C) Gene expression levels of each synovial sample after batch effect removal. (D) UMAP plot of synovial samples before batch effect removal. (E) UMAP [file 12967_2023_4173_MOESM2_ESM.docx]

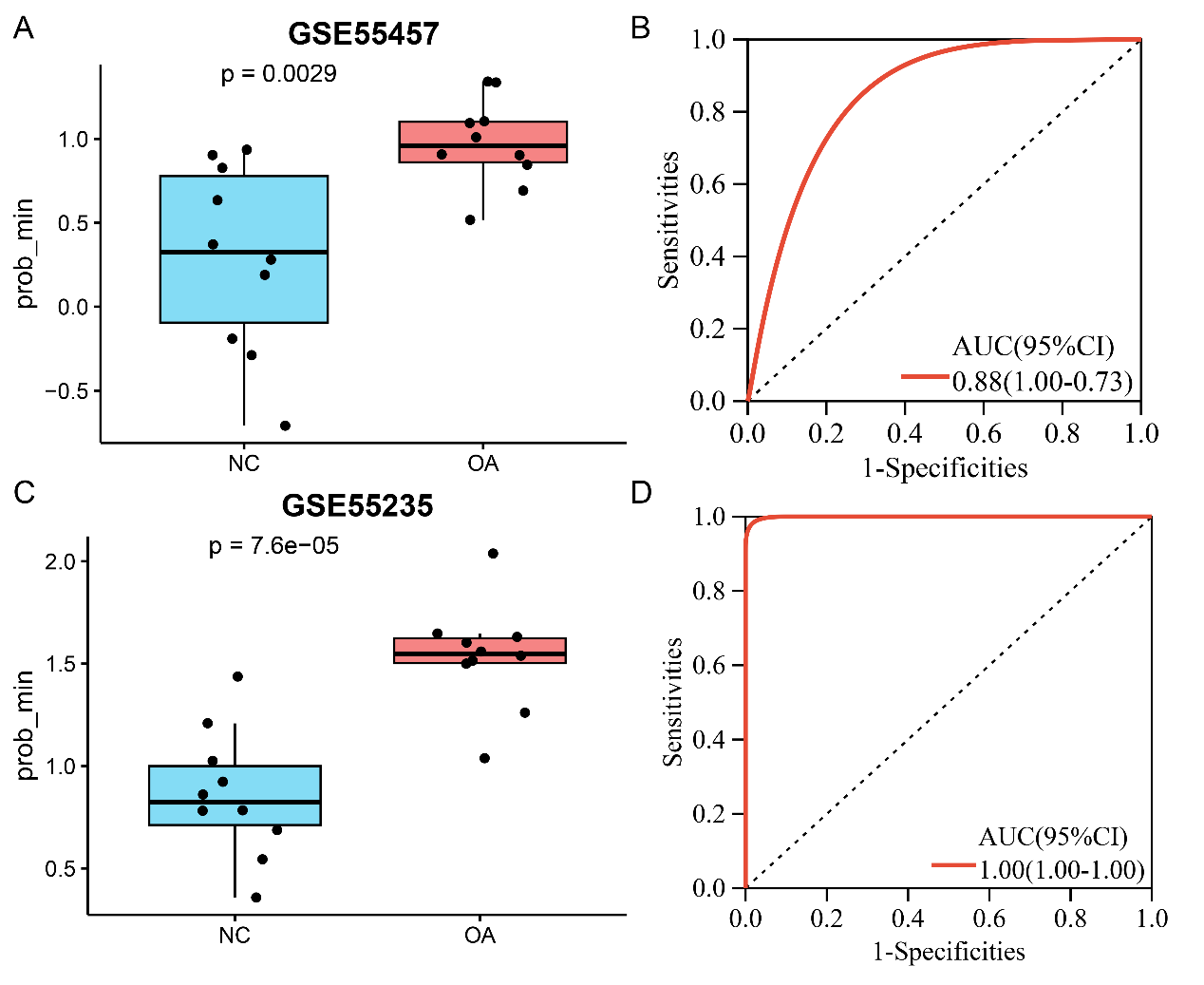


**Figure S1.** (A) OA probabilities (Prob-min) of Lasso-Cox model in testing set GSE55457. (B) ROC curve for the OA prediction model based on the expression levels of 6 m6A regulators in testing set GSE55457. (C) OA probabilities (Prob-min) of Lasso-Cox model in testing set GSE55235. (D) ROC curve for the OA prediction model based on the expression levels of 6 m6A regulators in testing set GSE55235.


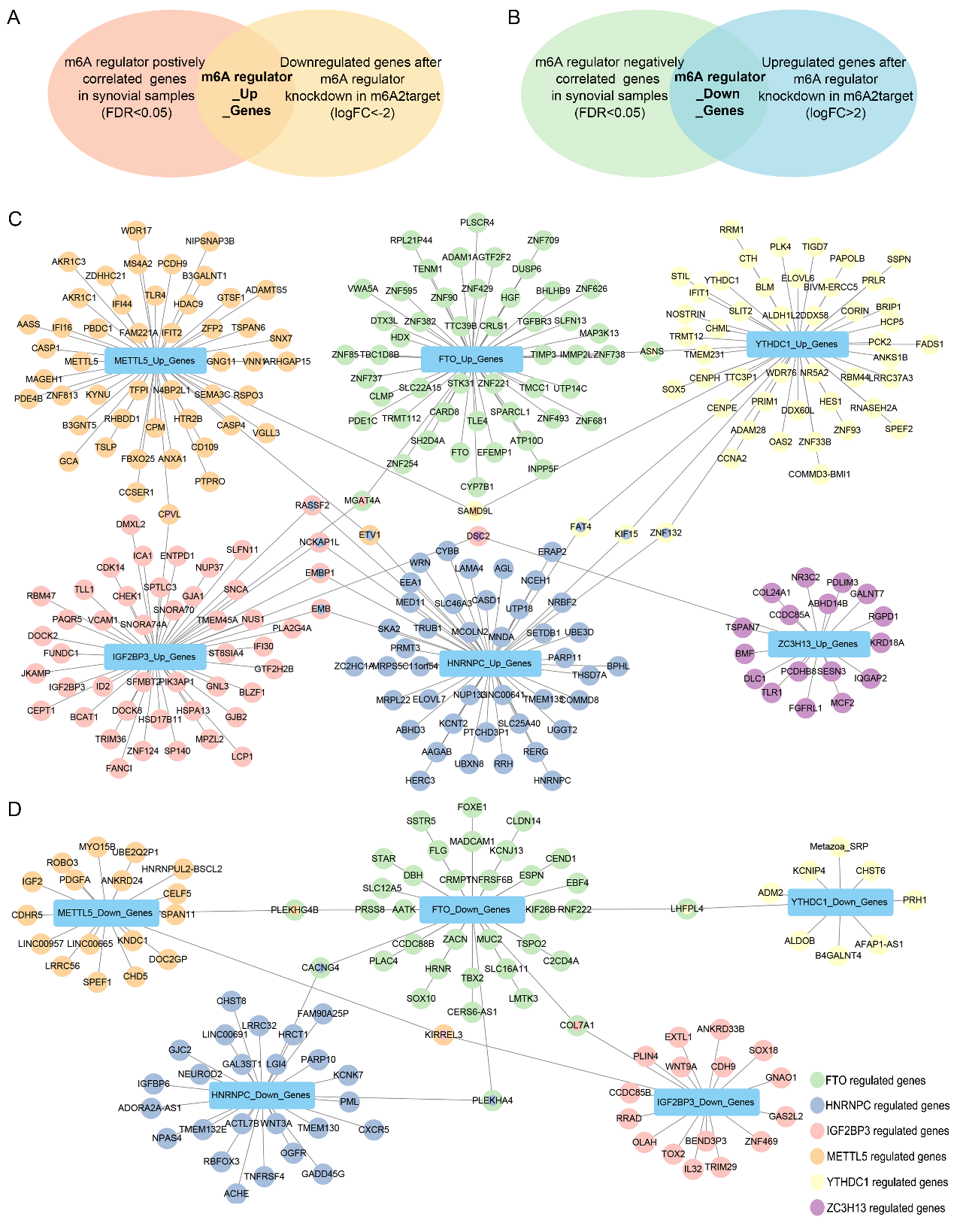


**Figure S2.** (A, B) Screening strategy of m6A up/down genes. (C) Illustration of METTL5-, FTO-, YTHDC1-, IGF2BP3-, HNRNPC-, and ZC3H13- upregulated genes. (D) Illustration of METTL5-, FTO-, YTHDC1-, IGF2BP3-, and HNRNPC- downregulated genes. Nodes in green, blue, pink, orange, yellow, and purple represent FTO-, HNRNPC-, IGF2BP3-, METTL5-, YTHDC1-, ZC3H13-regulated genes.


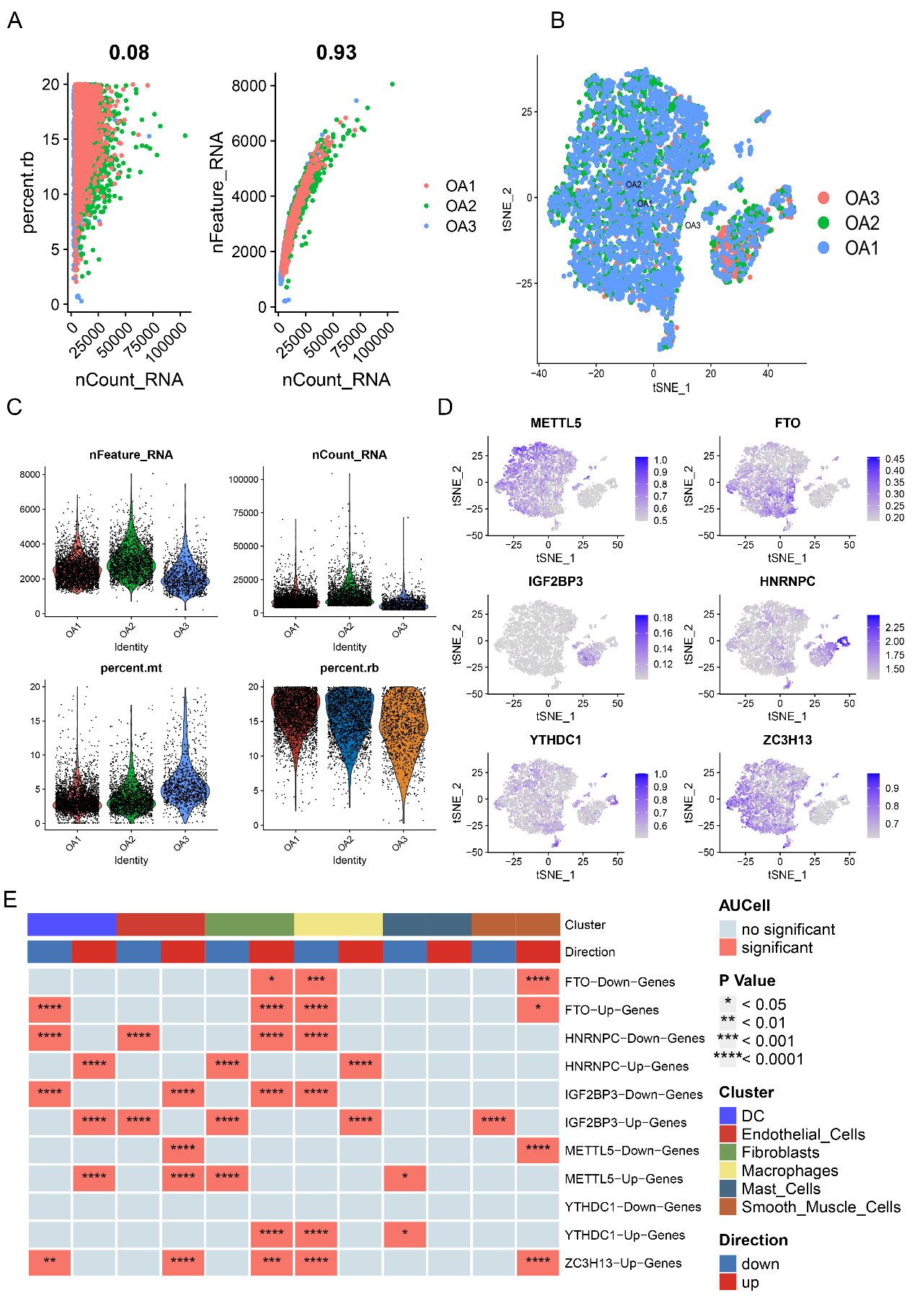


**Figure S3.** (A) Correlation between gene expression levels (nCount_RNA) and ribosome gene proportion (percent.rb), and correlation between gene expression levels (nCount_RNA) and gene numbers (nFeature_RNA) of synovium samples of of scRNA-seq dataset GSE152805. (B) t-SNE plot of synovial cells in each sample after batch effects removal with Harmony. (C) Gene numbers (nFeature_RNA), gene expression levels (nCount_RNA), mitochondria gene proportion (percent.mt), and ribosome gene proportion (percent.rb) in each synovium sample. Cells with either fewer than 200 expressed genes, more than 10,000 expressed genes, or over 20% UMIs derived from mitochondrial and ribosomal genome were excluded. (D)t-SNE plots of METTL5, FTO, IGF2BP3, HNRNPC, YTHDC1, and ZC3H13 expressions. (E) Heatmap of AUCell scores of core m6A regulators upregulated and downregulated genes in each cell cluster.


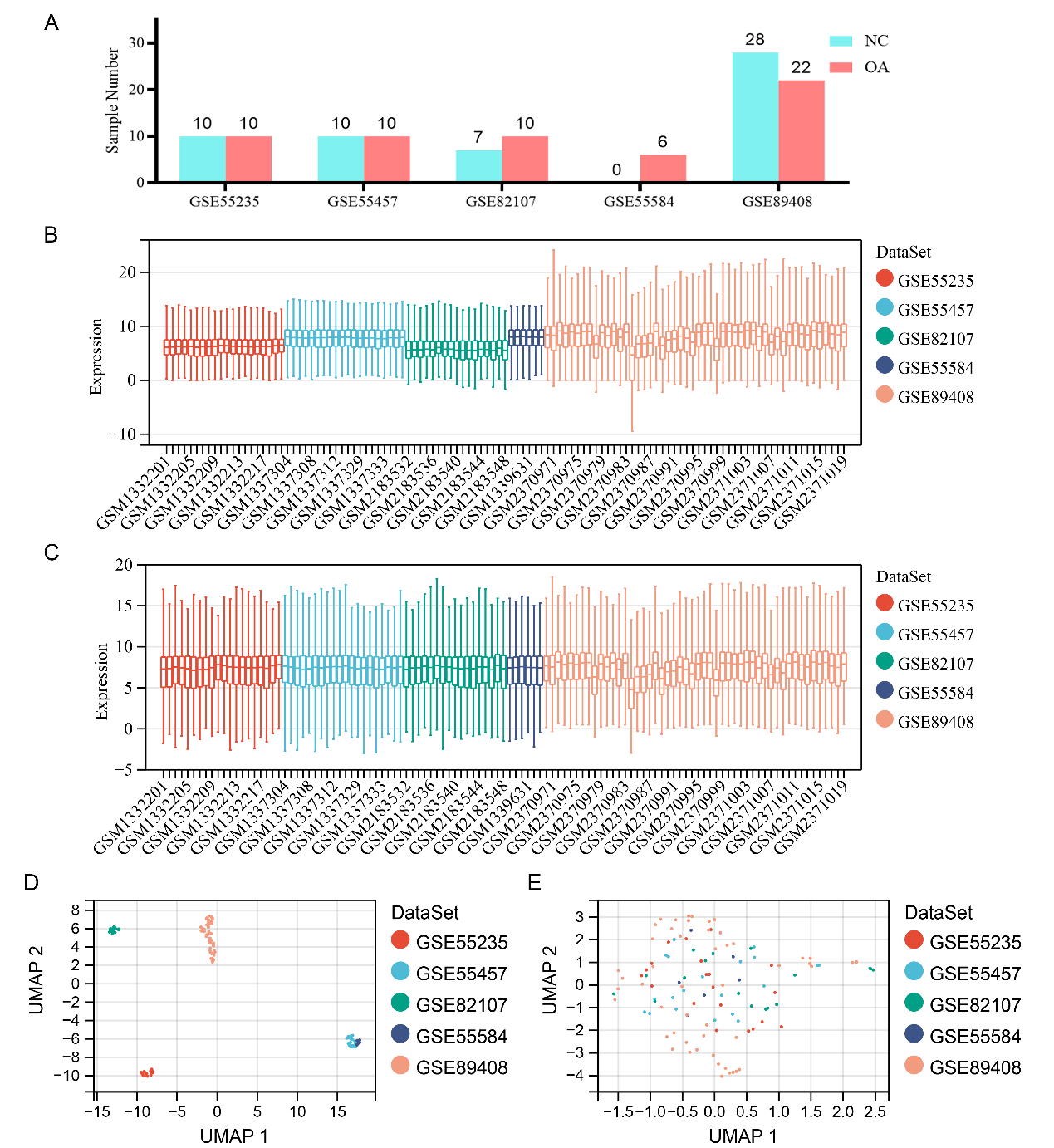


**Figure S4.** (A) Sample numbers of control and OA synovium in each public bulk RNA-seq dataset. (B) Gene expression levels of each synovial sample before batch effect removal. (C) Gene expression levels of each synovial sample after batch effect removal. (D) UMAP plot of synovial samples before batch effect removal. (E) UMAP plot of synovial samples after batch effect removal.


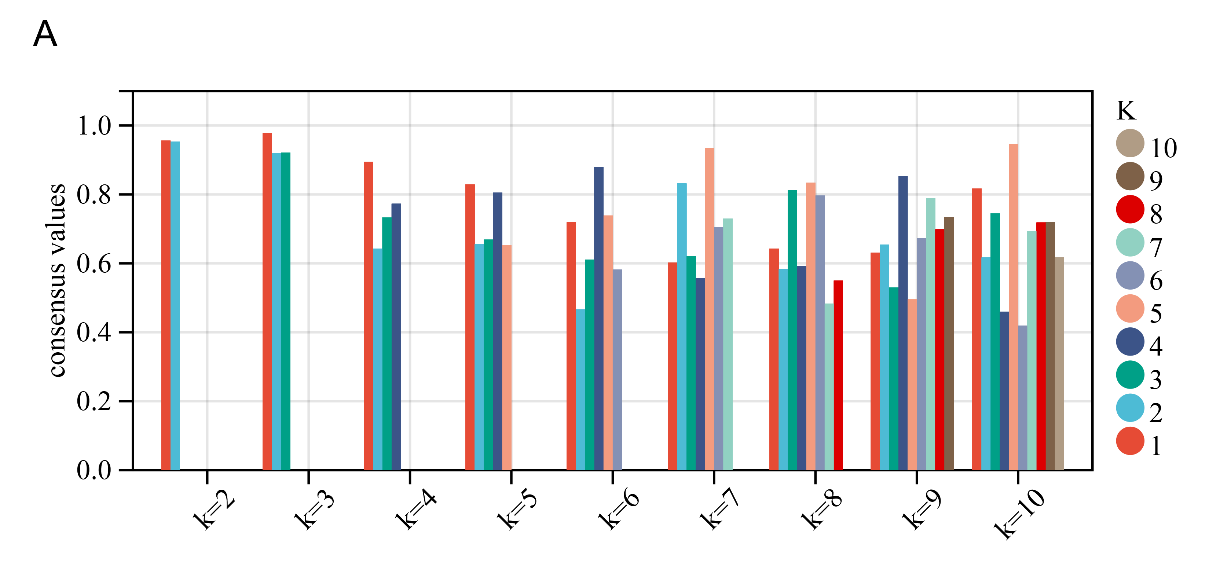


**Figure S5.** (A) Consensus values of different clustering numbers of synovial samples. When k=2, averaged cluster-consensus reached the highest


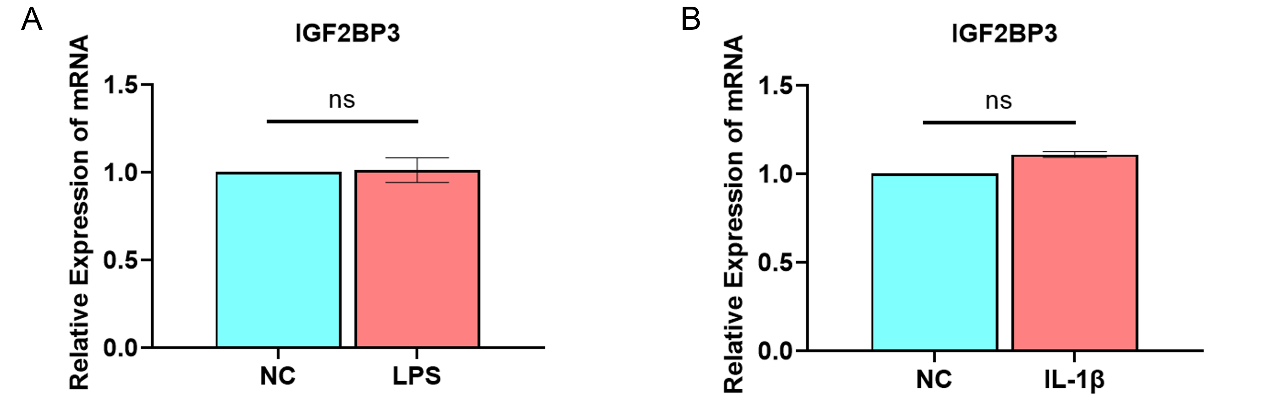


**Figure S6. (**A) qPCR analysis of IGF2BP3 in LPS treated BMDMs (n=3). (B) qPCR analysis of IGF2BP3 in IL-1β treated BMDMs (n=3). ns not significant.


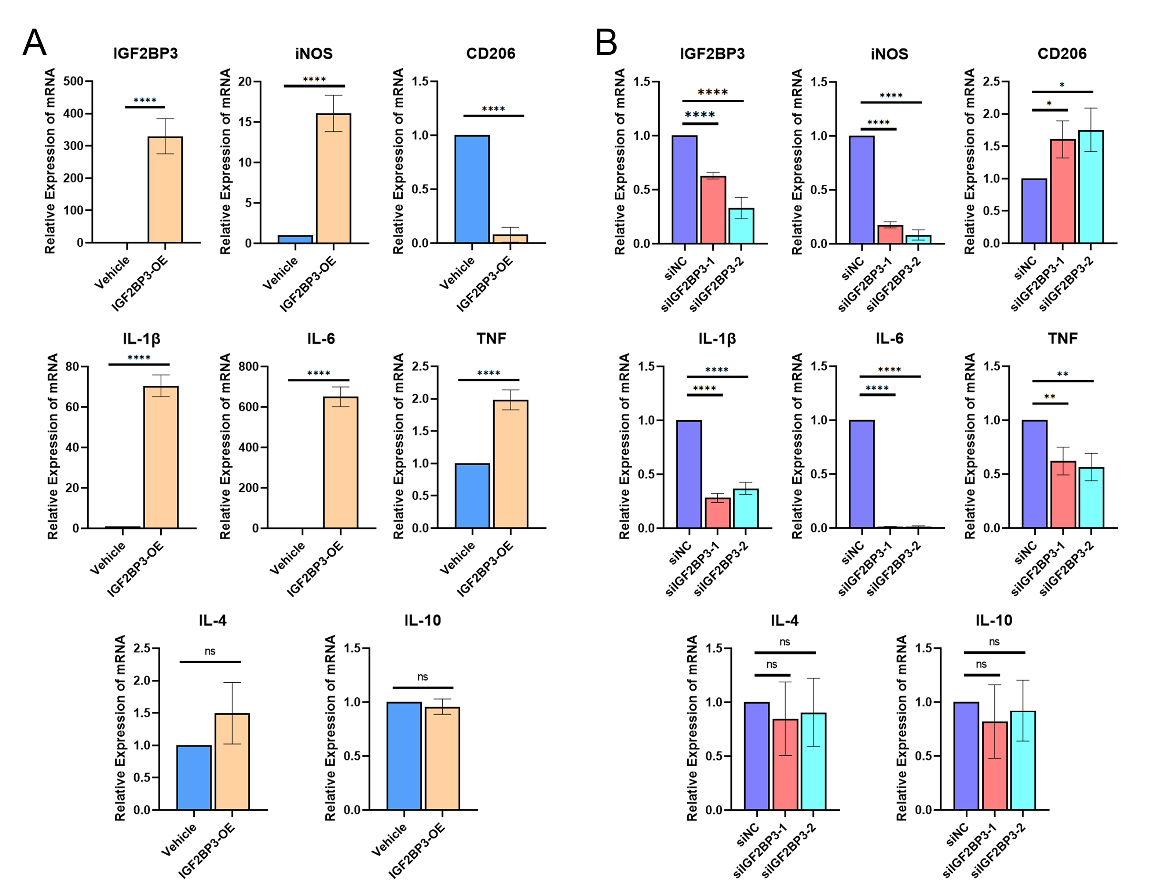


**Figure S7.** (A) qPCR assay of IGF2BP3 expression, polarization (iNOS, CD206), inflammation (IL-1β, IL-6, TNF) and anti-inflammation (IL-4, IL-10) genes in IGF2BP3 overexpressed BMDMs (n=3). (B) qPCR assay of IGF2BP3 expression, polarization (iNOS, CD206), inflammation (IL-1β, IL-6, TNF) and anti-inflammation (IL-4, IL-10) genes in IGF2BP3 knock down BMDMs (n=3). * *P* < 0.05, ***P* < 0.01, *****P* < 0.0001, ns not significant.
